# An evaluation of an educational intervention for improving concussion knowledge among medical students

**DOI:** 10.1371/journal.pone.0352810

**Published:** 2026-06-30

**Authors:** Josheil Boparai, Chris Compton, Ryan Murray, Cole Winsor, Ashley Stringer, Mitchell Fagan, Ben Collingwood, Roger Avery

**Affiliations:** Faculty of Medicine, Memorial University of Newfoundland and Labrador, St. John’s, Newfoundland and Labrador, Canada; Drexel University School of Biomedical Engineering Science and Health Systems, UNITED STATES OF AMERICA

## Abstract

**Background:**

There are significant gaps in concussion education in the undergraduate medical curriculum, contributing to poor concussion management in clinical practice.

**Objective:**

The purpose of the current study is to evaluate the effectiveness of an educational intervention for improving concussion knowledge among undergraduate medical students.

**Methods:**

An interventional pre-post study was conducted wherein participants first completed a pre-survey, followed by a didactic lecture about concussions and two post-surveys; one assessing short-term knowledge retention (within 48 hours of the lecture delivery) and long-term knowledge retention (within 2 weeks of the lecture delivery).

**Results:**

A one-way ANOVA showed a statistically significant difference in concussion knowledge scores between the three time points, F(2, 156) = 77.38, p < .001, with the mean score for the pre-lecture group being significantly lower than both the immediate and the two-week post-lecture groups (p < .001). There was no statistically significant difference between the immediate and two-week post-lecture groups (p = .975, 95% CI = [−.57,  .68]), suggesting knowledge retention over time.

**Conclusion:**

The improvement in concussion knowledge scores following a didactic lecture demonstrates the importance of the inclusion of teaching time dedicated to concussion education, which may in turn, contribute to improvements in clinical care and patient satisfaction.

## Introduction

Concussions are defined as traumatic brain injuries caused by direct or indirect injury to the head resulting in a functional disturbance which can manifest as mild, transient symptoms to extended periods of altered consciousness [1]. The true prevalence of concussion is unknown due to inconsistencies across study methods, as well as underreporting due to a lack of awareness and inadequate surveillance. An Ontario study reported an annual incidence of concussion at a mean of 1153 per 100 000 or greater than 1% of the population of Ontario per year [2]. Given the substantial prevalence of concussions, it is important for physicians to be equipped with a strong foundational knowledge of concussions.

Previous research has shown significant gaps in medical education on concussions. Although many published guidelines on concussion management exist, studies have shown that many physicians are not aware of the existence of such guidelines, do not implement their recommendations, and do not use standardized assessments of concussion [3–6]. This can result in unnecessary referrals to specialists for treatment [3] and poor-quality care resulting in prolonged or inadequate recovery [7].

The inadequate physician competence with concussions may be attributed to a paucity of concussion education in the undergraduate and postgraduate medical curriculums. For example, a recent scoping review examining undergraduate concussion education concluded that a significant proportion of medical students are not receiving any education about concussion throughout their entire undergraduate training [8]. In fact, a 2012 survey of Canadian medical schools found that 29% (4 out of 14) of universities were not dedicating any time to teaching about concussion [9]. Although a 5-year follow-up study showed significant improvement with 85% of surveyed medical schools (11 out of 13) having concussion-specific education in their curriculum [10], these findings should be interpreted with caution, as they do not represent all 18 Canadian medical schools. Additionally, this improvement does not evaluate the time, quality or consistency of the educational content, nor whether it translated into improved clinical competence. Furthermore, of the 11 schools that participated in both surveys, there was considerable variability in teaching time and approach, with only 7 (64%) reporting an increase in teaching time dedicated to concussion-specific material and 9 (82%) reporting an increase in general head-injury education [10]. While there is a promising increase in concussion education across Canadian medical schools, the adequacy of the content taught in concussion education and the effectiveness of existing teaching methods in improving concussion knowledge among medical trainees remains unclear. This current study aims to address this gap by evaluating the effectiveness of an educational intervention for improving concussion knowledge scores among undergraduate medical students.

## Materials and methods

An interventional pre-post study was conducted among a cohort of undergraduate medical student participants within Atlantic Canada. Participants completed a pre-concussion knowledge survey 48 hours before receiving an evidence-based didactic lecture on the topic of concussions. Participants completed a post-concussion knowledge survey within 48 hours of the lecture and again two weeks post-lecture. The pre- and post-concussion knowledge surveys were identical and are based on a survey our group adapted from Boggild & Tator [11] and used in an analysis of Canadian medical students and physicians’ concussion knowledge [12]. It has been modified to be more reflective of the latest consensus statement on concussion in sport [13] (See S1 Appendix). The survey included 27 items organized into 4 sections. Section 1 included demographic questions such as participant sex (male or female), gender identity (male, female, other (please specify), prefer not to specify), age, year of study (1, 2, 3, 4 or N/A), and personal history of concussions. Section 2 assessed students’ concussion knowledge with 12 questions which were a combination of multiple choice and select all that apply formats. Section 3 explored students’ concussion learning needs with questions about prior concussion teaching, preferred formats and resources. Section 5 was an opportunity for participants to provide open-ended feedback about the didactic lecture. The surveys were distributed via Qualtrics.

The didactic lecture about concussions was delivered using a Powerpoint presentation developed by the MUN Concussion-U Interest Group based on published evidence-based guidelines [14–16] with feedback from three sports medicine physicians (See S2 Appendix). Over 60 minutes, participants learned about the definition, epidemiology, pathophysiology, clinical evaluation, diagnoses, and management of concussion injuries. The lecture was delivered via a combination of virtual and in-person formats by members of the Concussion-U team using a standardized script of speaker notes to ensure consistency between sessions. Data was collected between March 13, 2023 – February 16, 2025. This study received ethics approval from the provincial Health Research Ethics Board (HREB) of Memorial University of Newfoundland and Labrador (MUN) (HREB# 20231426).

### Study population

Medical students enrolled in the Faculty of Medicine at Memorial University of Newfoundland and Labrador were eligible for participation in the study. To be included in the study, participants were required to attend the medical lecture as well as complete all three concussion knowledge surveys to be included in the study. Based on a population size of about 856 Atlantic Canadian medical students, a 95% confidence interval (z-score = 1.96), and a 10% margin of error, the target sample size for this study was 87 participants.

Participants were invited to participate via email solicitation and private class Facebook groups. Prior to participation, participants read and signed an informed consent form to confirm registration. Participants were offered the opportunity to enter a draw at the end of the study for a $50 visa gift card.

### Statistical analysis

Data was analyzed quantitatively using IBM SPSS Statistics Version 29. The outcome measure was mean concussion knowledge scores on the concussion knowledge surveys. A one-way ANOVA was conducted to compare pre- and post-lecture survey scores to determine the effectiveness of the lecture on medical students’ short-term knowledge retention (within 48 hours of intervention) and long-term knowledge retention (two weeks post-intervention). Homogeneity of variance was assessed using Levene’s test. Alpha was set at *p* < 0.05.

## Results

### Demographics

77 undergraduate medical students registered to participate over three years (2022–2024). However, only 57 participants completed all three concussion knowledge surveys. Of these, four participants used incorrect participant IDs to complete the final survey, and their data could not be used as it could not be reliably determined which participant the data was associated with. Thus, the final sample consisted of 53 undergraduate medical students, of which 48 completed the demographic survey (n = 31 females, 64.58%). Students from year 1–4 participated, with most participants in year 2 (MS2) (n = 33), followed by year 1 (MS1) (n = 13), year 3 (MS3) (n = 1), and year 4 (MS4) (n = 1). See Table 1 for details regarding the characteristics of included participants.

**Table 1:** Characteristics of included participants in the cross-sectional analysis.

	Number of Participants (n, %)
**Gender Identity**	
**Male** (n, %)	17 (35.42%)
**Female** (n, %)	31 (64.58%)
**Year of Medical School**	
**MS1** (n, %)	13 (27.08%)
**MS2** (n, %)	33 (68.75%)
**MS3** (n, %)	1 (2.08%)
**MS4** (n, %)	1 (2.08%)
**Seen a concussion patient (acute phase)**	6 (12.5%)
**Seen a patient with post-concussion syndrome**	6 (12.5%)
**Personal history of concussion**	13 (27.08%)
**Number of previous concussions**	
**0**	35
**1-2**	10
**3-4**	2
**≥ 5**	1
**Preferred Format for physician learning material**	
**Lecture**	33 (68.75%)
**Seminar/Workshop**	24 (50%)
**Clinical Experience**	45 (93.75%)
**Pamphlet**	1 (2.08%)
**Resources Used for Concussion Information**	
Google	33 (68.75%)
Up To Date	14 (29.17%)
Wikipedia	6 (12.5%)
PubMed	26 (54.17%)
**Concussion Interest** (1 = Not at all, 10 = very much)	
1-4	1 (2.08%)
5-7	12 (25%)
8-10	35 (72.92%)
**Baseline Knowledge of Concussion** (1 = inadequate; 10 = adequate)^a^	
1-4	35 (72.92%)
5-7	13 (27.08%)
8-10	0
**Concussion Learning in UGME**	
Clerkship or Shadowing Experience	2 (4.17%)
Interest Group or Seminar	9 (18.75%)
Lecture	1 (2.03%)
Other	9 (18.75%)
Never or Unable to Recall	29 (60.42%)

^**a**^Baseline knowledge of concussion was assessed subjectively based on participants’ self-reported ratings as seen in the table above and objectively using their scores on the concussion knowledge survey.

### Effect of a didactic lecture on medical students’ concussion knowledge

A one-way ANOVA was conducted to compare concussion knowledge scores across three time points: pre-lecture, immediate post-lecture (within 48 hours), and two weeks post-lecture. There was a statistically significant difference in concussion knowledge scores between time points, F(2, 156) = 77.38, p < .001.

**Fig 1:**
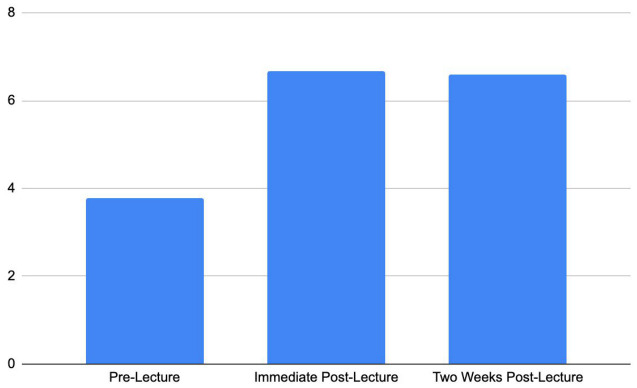
Concussion knowledge scores across time points. This bar graph shows the concussion knowledge scores across three time points: pre-lecture, immediate post-lecture (within 48 hours) and two weeks post-lecture.

Tukey’s HSD test for multiple comparisons was done post-hoc and showed that the mean score for the pre-lecture group (M = 3.79, SD = 1.28) was significantly lower than both the immediate post-lecture group (M = 6.66, SD = 1.49, p < .001, 95% CI = [−3.49, −2.44]) and the two-week post-lecture group (M = 6.60, SD = 1.29, p < .001, 95% CI = [−3.43, −2.18]). See Fig 1 with a visual representation of change in groups’ scores over time. There was no statistically significant difference between the immediate and two-week post-lecture groups (p = .975, 95% CI = [−.57,  .68]), suggesting knowledge retention over time.

Effect size analyses showed a large effect of time on concussion knowledge, with eta-squared = .498, indicating that approximately 50% of the variance in scores was accounted for by the intervention.

Tests of homogeneity of variances were non-significant (Levene’s test: p > .05), supporting the assumption of equal variances across groups.

## Discussion

This study investigated the effectiveness of an educational intervention for improving concussion knowledge scores among undergraduate medical students. The results of this study show that a didactic lecture on concussion improved concussion knowledge scores among undergraduate medical students, in the short-term (within 48 hours) and long-term (within 2 weeks). These findings align with prior studies conducted with resident physicians [17], as well as students in athletic training and medical dietetics [18], which show a measurable improvement in knowledge after a didactic lecture. This adds supporting evidence that there is value in increasing the amount of formal didactic concussion education in medical school curriculums. Despite the improvement, overall scores still remained suboptimal following the intervention with an improvement from 3.79 to 6.66 and 6.60 out of a possible 12 points, on average. This is consistent with previous findings that suggest low baseline concussion literacy [12], which reiterates the need for improvement in concussion education.

Previous research with the same survey without the current modifications has shown that concussion knowledge scores were significantly higher among clerkship students than pre-clinical students [12], suggesting that most concussion learning was taking place in clinical settings. Given that most study participants were pre-clinical learners in the present study, we cannot compare knowledge scores between pre-clinical and clinical learners. However, our results do demonstrate a substantial improvement in concussion knowledge among pre-clinical learners. If implemented more systematically among pre-clinical medical curriculums, our study demonstrates that didactic teaching on concussions among pre-clinical learners shows promise in augmenting baseline knowledge and enhancing clinical learning. Further research should aim at recruiting more clinical learners including those in medical clerkship and residency to better analyze the effect of the intervention among these cohorts with more clinical experience. Replication of these results across various medical schools would help determine the generalization of these findings to the target population, as each medical school has their own curriculum with different emphasis on concussion education.

Concussions are the most common type of traumatic brain injury, accounting for approximately 80% to 95% of such injuries [19]. According to Statistics Canada [20], 1.6% of Canadians aged 12 years or older reported at least one concussion in 2019. Given the substantial prevalence of concussions and physicians being the most frequent point of contact following a concussion, there is a significant need for the implementation of concussion education in the undergraduate medical curriculum. 73.8% of Canadians with a concussion were diagnosed by a medical doctor or nurse practitioner [20]. However, prior research has demonstrated gaps in evidence-based practices and the delivery of care, particularly among family doctors and emergency physicians [17,21,22]. Many students report their first exposure to concussion learning to be from didactic lectures followed by their emergency department clerkship [17]. However, Gowdy and Heron [21] conclude inadequate adherence to established guidelines among emergency department physicians which may result in medical students learning incorrect concussion management and further contribute to lapses in patient care. As a result, concussion education in undergraduate medical education is important to ensure that future physicians are well-equipped to serve the diverse needs of their patient populations.

## Limitations

This study is not without limitations. The small sample size (n = 53) consisted of undergraduate medical students attending Memorial University of Newfoundland, most of which were first- or second-year students, which does not represent all medical students across Canada. This limits generalizability to the target population of Canadian medical students. Study participation was voluntary and as such, students who chose to participate may have had a pre-existing interest in concussions or medical education or personal experience with concussions, potentially introducing selection bias. Furthermore, the study was available to students in all training levels from year 1 to year 4, however, analyses were not stratified according to training level due to the small sample size. Similarly, although data was collected regarding participants’ prior exposure to concussion education, the subsample sizes were too small to compare how exposure influenced self-reported baseline concussion knowledge or response to the educational intervention. Additionally, the answers to the pre and post-tests were assessed in an all-or-none manner. Questions were only marked as correct if they selected all the correct options and no incorrect options, thus potentially misrepresenting the depth of knowledge. Furthermore, our study assessed long-term knowledge retention at a two-week interval and while this showed a sustainable increase, future studies would benefit from incorporating lengthier follow-up timepoints, such as during students’ yearly progression in medical training, to better evaluate retention as learners progress through medical school. Additionally, collecting demographic variables such as age and race or ethnicity may help identify differences in knowledge acquisition and retention, thereby strengthening the interpretability and generalizability of findings.

## Conclusions

In this study, we demonstrated that a didactic lecture on concussion education improved concussion knowledge that was retained in follow up. Although improved with the intervention, overall concussion literacy remained quite low in this cohort of medical learners. These findings demonstrate the importance and effectiveness of including dedicated teaching time to concussion education in the medical curriculum. Future studies should investigate the impact of this intervention across Canadian medical schools and various stages of undergraduate medical training to further elucidate differences in effectiveness, identify optimal timing for implementation, and assess generalizability of the intervention.

## Supporting information

S1 Appendix Concussion Knowledge Survey. (DOCX)

S2 Appendix Concussion Educational Intervention. (PPTX)
