# Supplementary material for: An evaluation of an educational intervention for improving concussion knowledge among medical students
Source: PLoS One. 2026 Jun 30;21(6):e0352810. doi: 10.1371/journal.pone.0352810 (PMC13318013; doi:10.1371/journal.pone.0352810)
Supplement: S1 Appendix — (DOCX) [file pone.0352810.s001.docx]

**S1 Appendix**

**Concussion Knowledge Survey**

Answers highlighted in red

**Part 1: ID & Demographic**

1. What is your sex assigned at birth? Male, Female
2. What is your current gender identity? *Male, Female, Other (please specify), Prefer not to specify*
3. What is your current age? *(Fill in)*
4. Are you currently an undergraduate medical student? *Yes, No*
5. What year of undergraduate education are you currently in? *1, 2, 3, 4, N/A*
6. In the past, have you ever been diagnosed with a concussion? *Yes or no?*
7. If you answered yes to the previous question, how many concussion(s) have you experienced? *1, 2-5, more than 5*
8. If you answered yes to the previous question (7), how did your concussion(s) occur? Please select all that apply. *Work related, Motor Vehicle Accident, Sport or recreational activity, Fall, Other*

**Part 2: Knowledge questions about concussions**

1. What is the best definition of concussion? Select the best answer.
   a. Loss of consciousness for <5 mins after an impact to the head
   b. A complex pathophysiological process affecting brain function, induced by traumatic biochemical forces transmitted to the head
   c. A structural brain injury caused by mild traumatic force that transiently decreases cerebral blood flow
2. Is a concussion a brain injury? Select the best answer.
   a. No, as there is no abnormality seen on standard structural neuroimaging
   b. No, as symptoms are only psychological in nature
   c. Yes, as there is a functional disturbance that cannot be seen on standard neuroimaging
   d. Yes, as there is structural abnormality seen on standard neuroimaging
3. Which one of the following is true?
   a. A period of unconsciousness is necessary for the diagnosis of a concussion
   b. Over 2/3 of all concussions involve loss of consciousness (LOC)
   c. 1/3 to 2/3 of all concussions involve loss of consciousness (LOC)
   d. Less than 1/3 of all concussions involve loss of consciousness (LOC)
4. Which of the following is a sign or symptom of a concussion? Select all that apply.
   a. Headache

b. Hemiparesis

c. Dizziness

d. Confusion

e. Fixed dilated pupil

f. Nausea and/or Vomiting

g. Vertigo

h. Amnesia

i. Tinnitus

j. Emotional or personality changes

k. Papilledema

l. Intention tremor

m. Fatigue

n. Temporary loss of consciousness

o. Prolonged Coma

1. How many symptoms of a concussion are required to diagnose a concussion?
2. One or more symptoms
3. Three or more symptoms
4. Five or more symptoms
5. Which of the following are red flags requiring immediate referral to an emergency department? Select all that apply.
6. Neck pain or tenderness
7. Double vision
8. Balance disturbance
9. Tinnitus
10. Repeated vomiting
11. Weakness or tingling on extremities
12. Severe or worsening headache
13. Seizures or convulsions
14. Restlessness, agitation, or combative behavior
15. Slowed processing speed
16. Deteriorating level of consciousness
17. Fixed dilated pupil
18. Refusal to eat or drink in children
19. Which of the following is true regarding the mechanism of concussion?
    a. Direct physical contact to the head is necessary to sustain a concussion
    b. Localized damage to the brainstem is the cause of a concussion
    c. Localized damage to the prefrontal cortex is the cause of a concussion
    d. Localized damage to the hippocampus is the cause of a concussion
    e. A whiplash effect to the brain caused by an impact to any part of the body may cause a concussion
20. Is baseline testing required for proper post-concussive care?
    a. Yes, baseline testing is required for proper post-concussive care
    b. No, baseline testing is not required for proper post-concussive care
    c. Unsure, the literature on baseline testing for proper post-concussive care is inconclusive
21. What is the appropriate management of concussion? Select all that apply.
    a. Every concussed individual should see a physician
    b. A concussed player can return to play in the same game or practice if examined by a physician
    c. A stepwise increase in activity guided by symptoms
    d. Physical rest is always recommended after a concussion
    e. Mental rest is always recommended after a concussion
    f. Signs and symptoms should be monitored for increasing severity
    g. Full neurological exam at initial assessment is recommended
    h. The standard mini mental status exam at initial assessment as an adequate cognitive test for concussion
    i. MRI of the brain is mandatory
    j. CT of the brain is mandatory
22. What is recommended regarding rest in the initial management of concussion?
23. 24-48 hours of relative physical and cognitive rest.
24. 24-48 hours of complete physical and cognitive rest.
25. 48-72 hours of complete physical and cognitive rest.
26. Physical and cognitive rest until complete recovery.
27. Physical and cognitive rest are not recommended.

11. Which of the following are risk factors for persistent post-concussion symptoms? Select all that apply

1. Nose bleed
2. Increased severity of initial symptoms
3. Female sex
4. Adolescence
5. Poor nutrition in acute recovery phase
6. Repeated concussions over time
7. Previous history of migraines
8. Previous history of anxiety or depression
9. Previous history of ADHD or learning disability
10. Being hit on the left side of the head

12. Which of the following are common persistent post-concussion symptoms? Select all that apply.

1. Headache
2. Nausea/vomiting
3. Vertigo
4. Fatigue
5. Impaired concentration
6. Confusion
7. Depression
8. Anxiety
9. Sleep disturbances
10. Aggression

**Part 3: Learning needs about concussions:**

1. Up to this point in your undergraduate medical education, how did you learn about concussions? Select all that apply.
   *Lecture, problem based learning, seminar, Interest Group, Shadowing/Observership, Clerkship rotation: ER, Clerkship rotation: Family medicine, Clerkship rotation: Pediatrics, Other Clerkship rotation, Other, Never, I can’t remember*
2. To date, have you seen a patient with:
   *Concussion in the acute phase? Yes, No, I don’t know
   Post-Concussive syndrome? Yes, No, I don’t know*
3. How would you self-rank your knowledge about concussions?
   *Inadequate (1) -> Adequate (10)*
4. What resource would you most likely use to find information about concussions?
   *Google, Wikipedia, Up-to-date, Textbook, Pubmed, an agency website, Parachute.ca, Published guidelines, other*
5. Are concussions something you want to learn more about as part of your medical curriculum?
   *Not at all (1) -> Very much (10)*
6. What is your preferred format for physician learning material?
   *Pamphlet, Letter, Seminar/Workshop, Lecture, Informational email, Clinical experience, Other (please specify)*

**Part 4: General Feedback**

1. Please provide any general comments or feedback that you have about the lecture presentation.

*Text box*
