# Supplementary material for: An evaluation of an educational intervention for improving concussion knowledge among medical students
Source: PLoS One. 2026 Jun 30;21(6):e0352810. doi: 10.1371/journal.pone.0352810 (PMC13318013; doi:10.1371/journal.pone.0352810)
Supplement: S2 Appendix — (PPTX) [file pone.0352810.s002.pptx]

## Slide 1
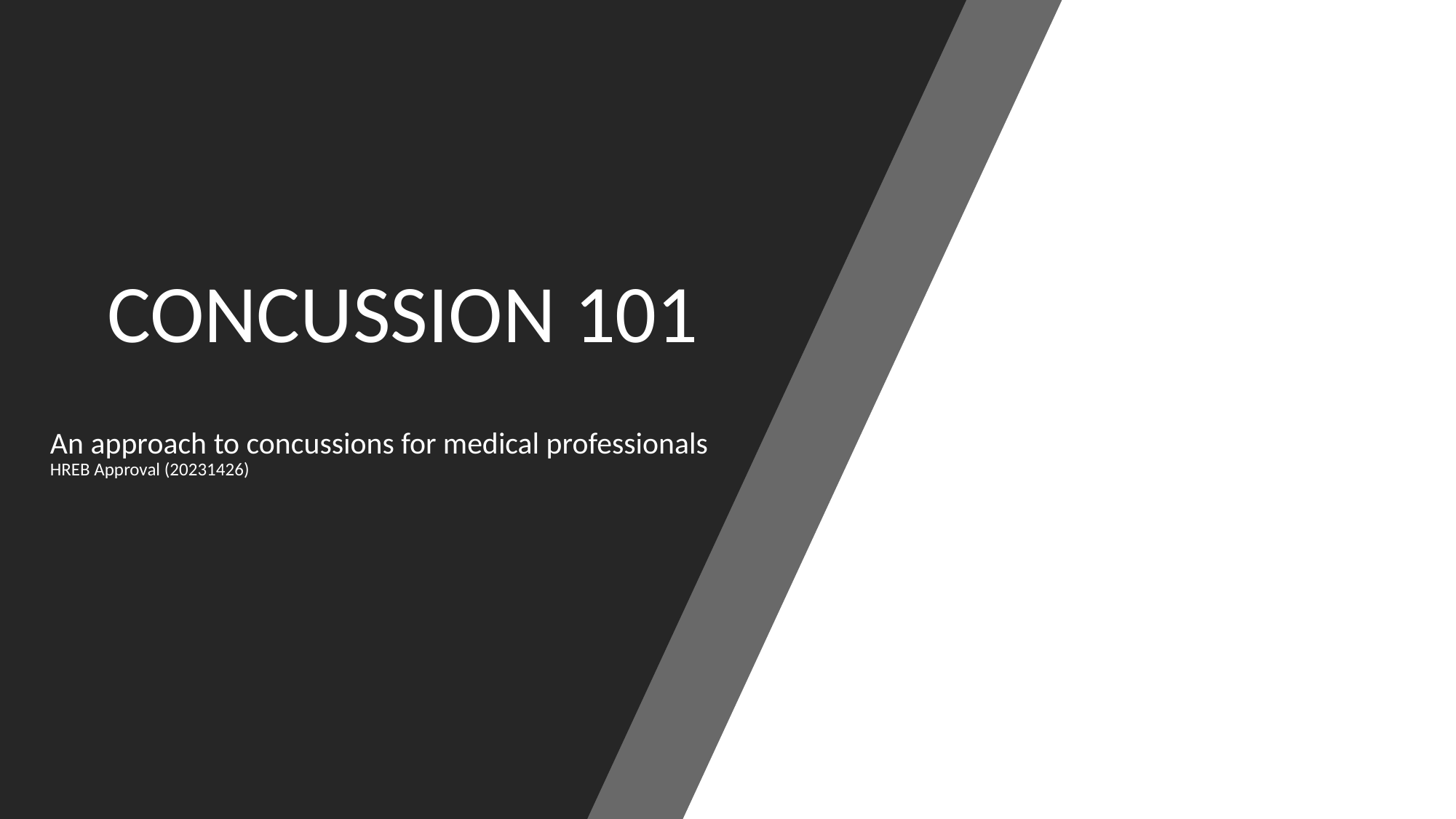

# CONCUSSION 101
An approach to concussions for medical professionals
HREB Approval (20231426)

## Slide 2
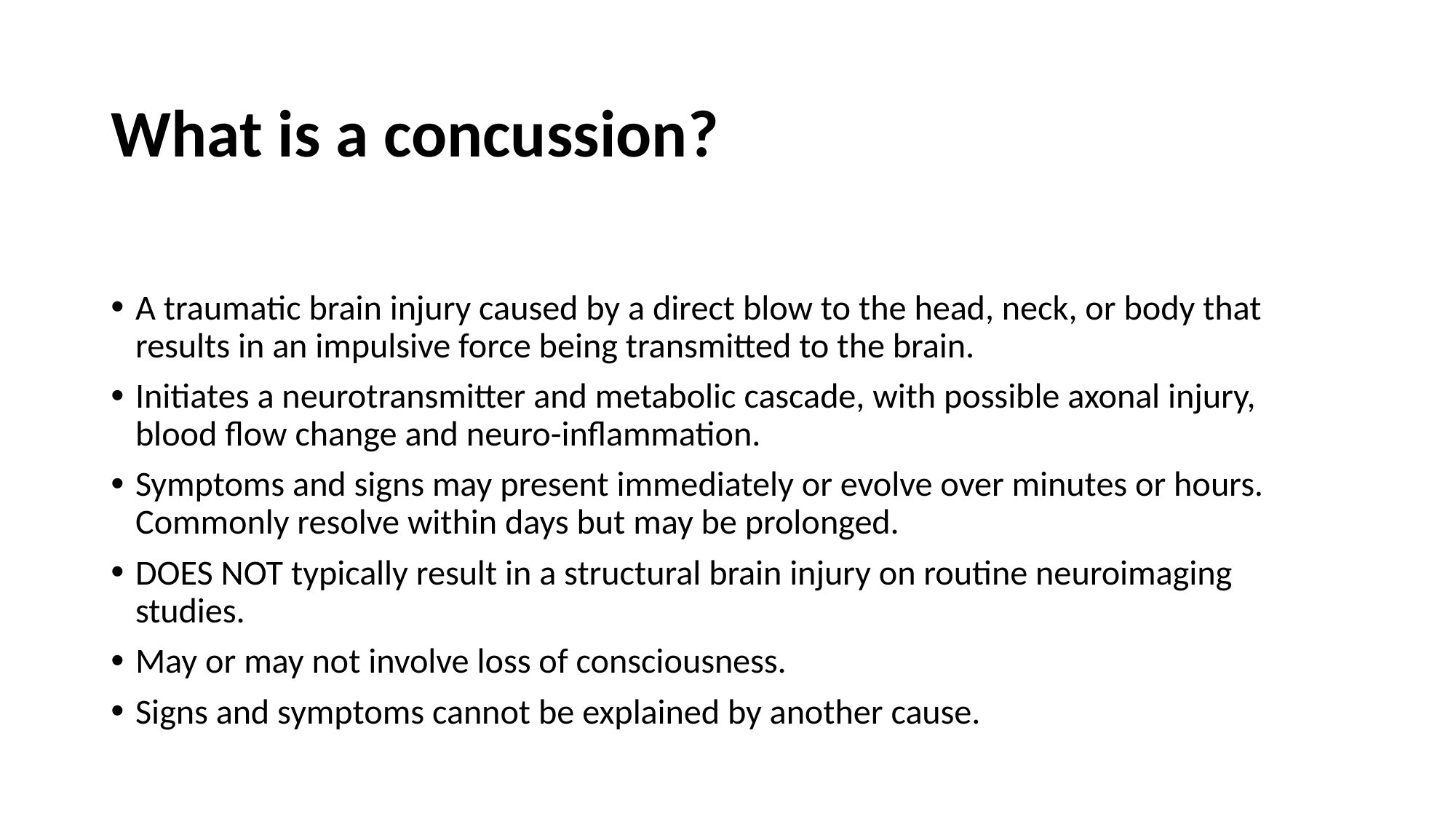

# What is a concussion?
A traumatic brain injury caused by a direct blow to the head, neck, or body that results in an impulsive force being transmitted to the brain.
Initiates a neurotransmitter and metabolic cascade, with possible axonal injury, blood flow change and neuro-inflammation.
Symptoms and signs may present immediately or evolve over minutes or hours. Commonly resolve within days but may be prolonged.
DOES NOT typically result in a structural brain injury on routine neuroimaging studies.
May or may not involve loss of consciousness.
Signs and symptoms cannot be explained by another cause.

## Slide 3
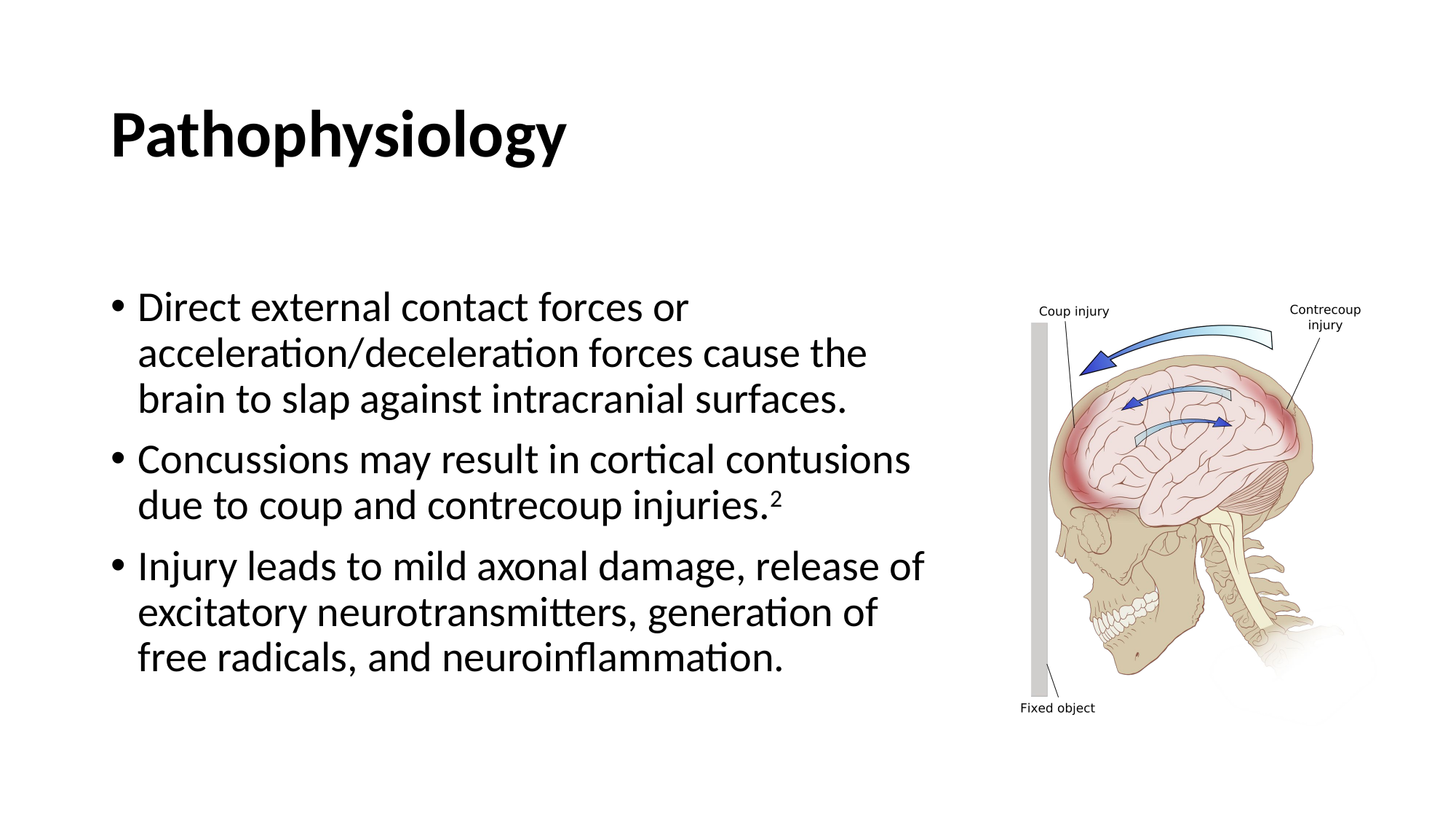

# Pathophysiology
Direct external contact forces or acceleration/deceleration forces cause the brain to slap against intracranial surfaces.
Concussions may result in cortical contusions due to coup and contrecoup injuries.2
Injury leads to mild axonal damage, release of excitatory neurotransmitters, generation of free radicals, and neuroinflammation.

## Slide 4
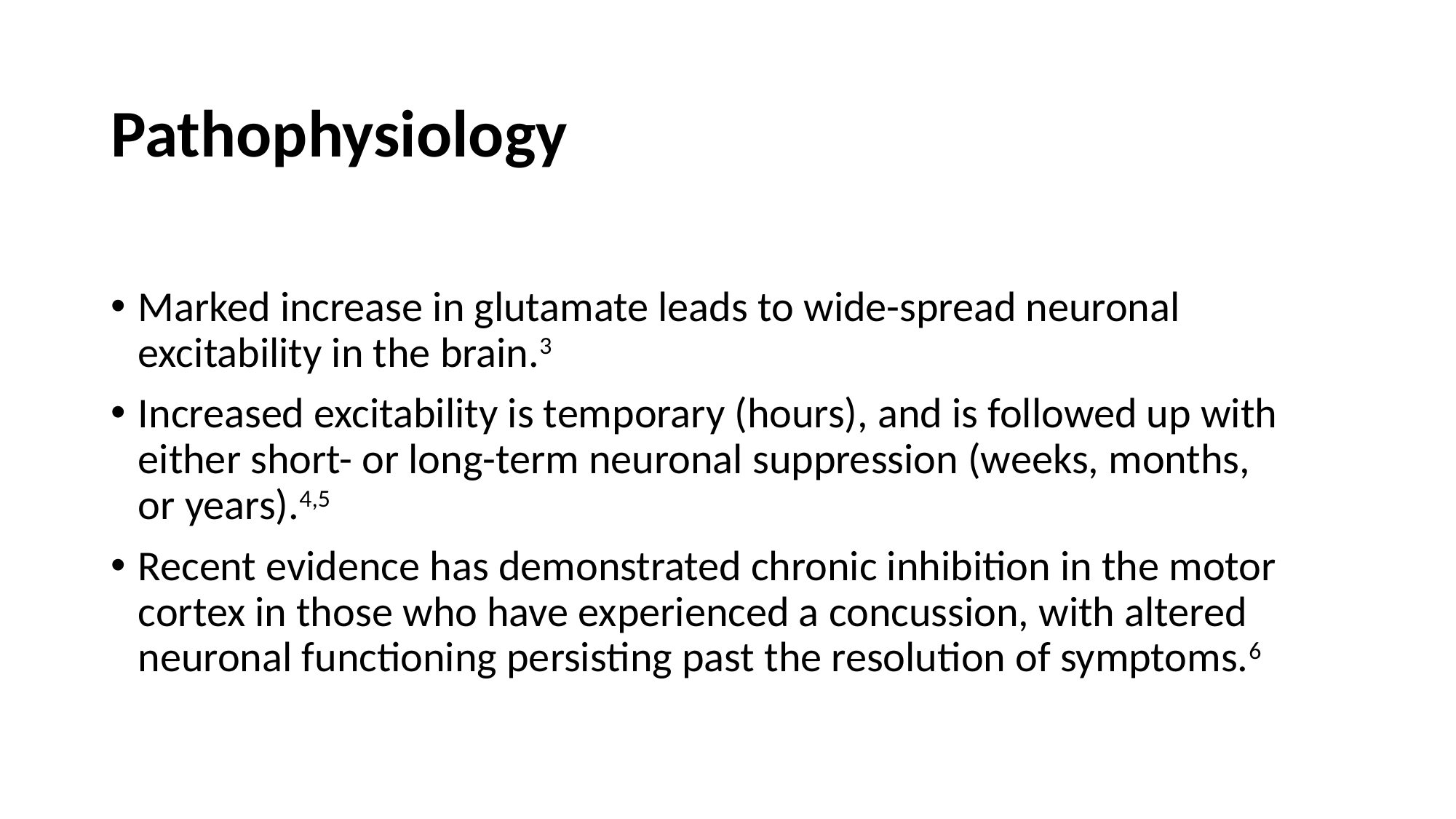

# Pathophysiology
Marked increase in glutamate leads to wide-spread neuronal excitability in the brain.3
Increased excitability is temporary (hours), and is followed up with either short- or long-term neuronal suppression (weeks, months, or years).4,5
Recent evidence has demonstrated chronic inhibition in the motor cortex in those who have experienced a concussion, with altered neuronal functioning persisting past the resolution of symptoms.6

## Slide 5
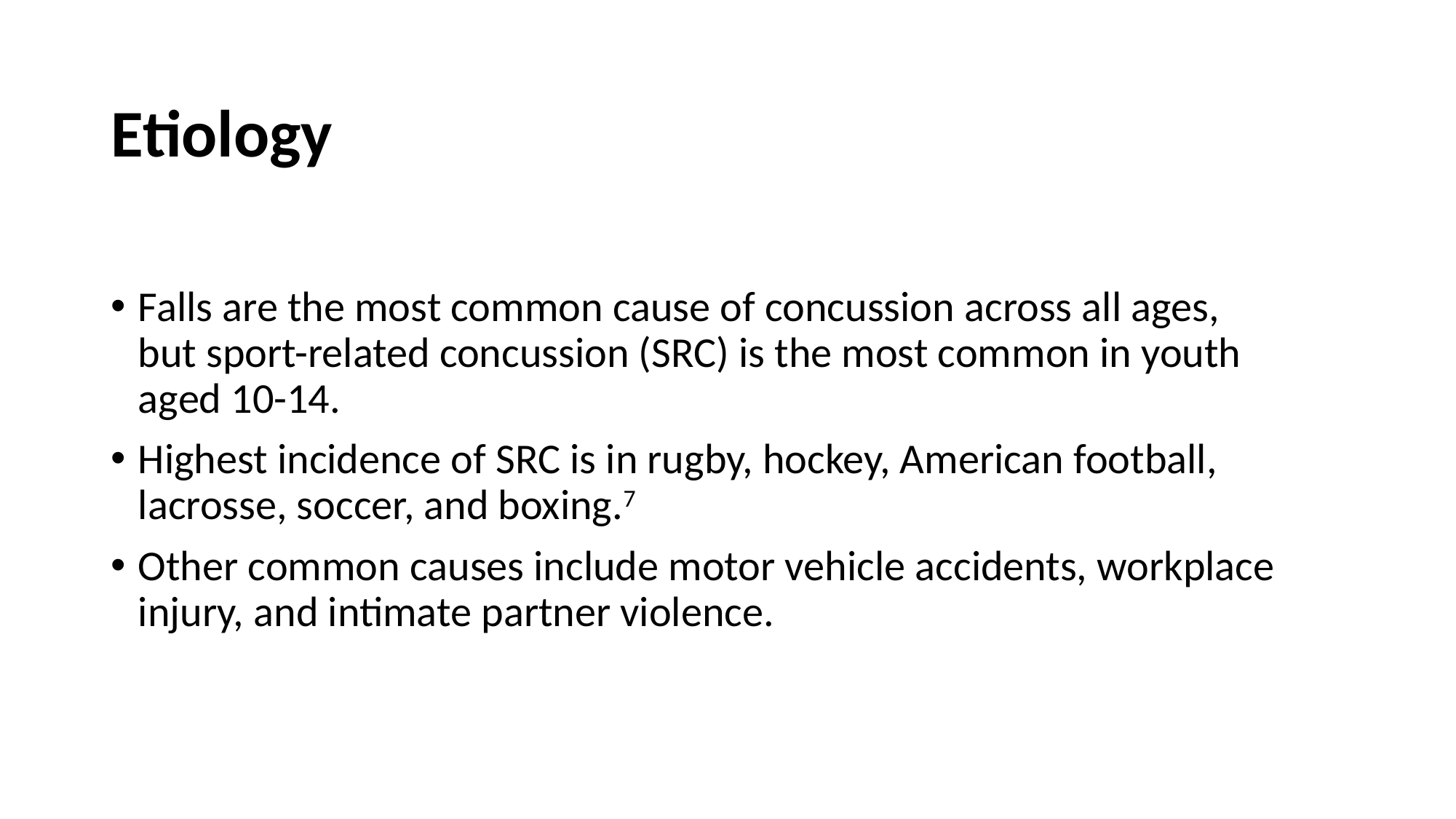

# Etiology
Falls are the most common cause of concussion across all ages, but sport-related concussion (SRC) is the most common in youth aged 10-14.
Highest incidence of SRC is in rugby, hockey, American football, lacrosse, soccer, and boxing.7
Other common causes include motor vehicle accidents, workplace injury, and intimate partner violence.

## Slide 6
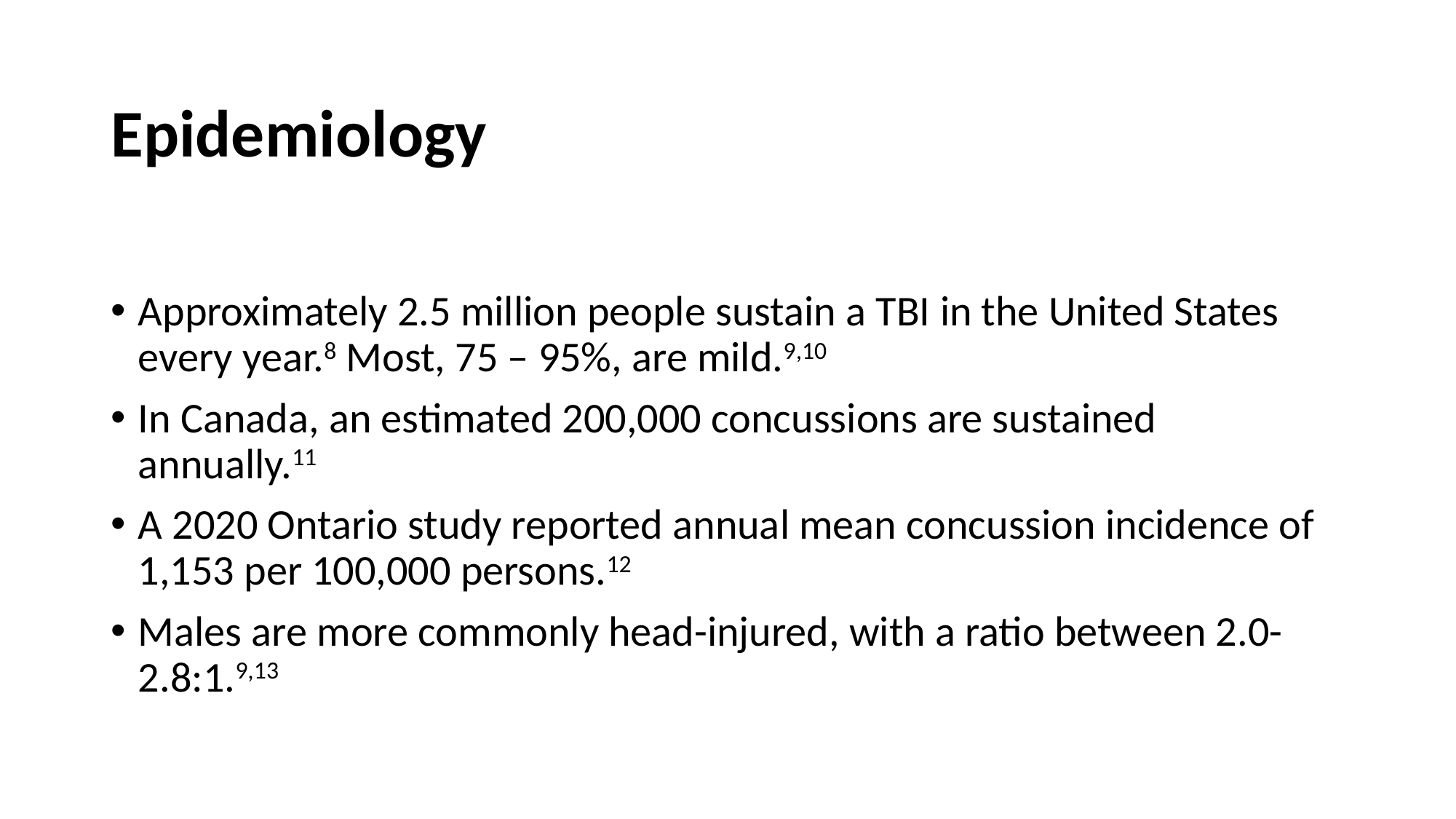

# Epidemiology
Approximately 2.5 million people sustain a TBI in the United States every year.8 Most, 75 – 95%, are mild.9,10
In Canada, an estimated 200,000 concussions are sustained annually.11
A 2020 Ontario study reported annual mean concussion incidence of 1,153 per 100,000 persons.12
Males are more commonly head-injured, with a ratio between 2.0-2.8:1.9,13

## Slide 7
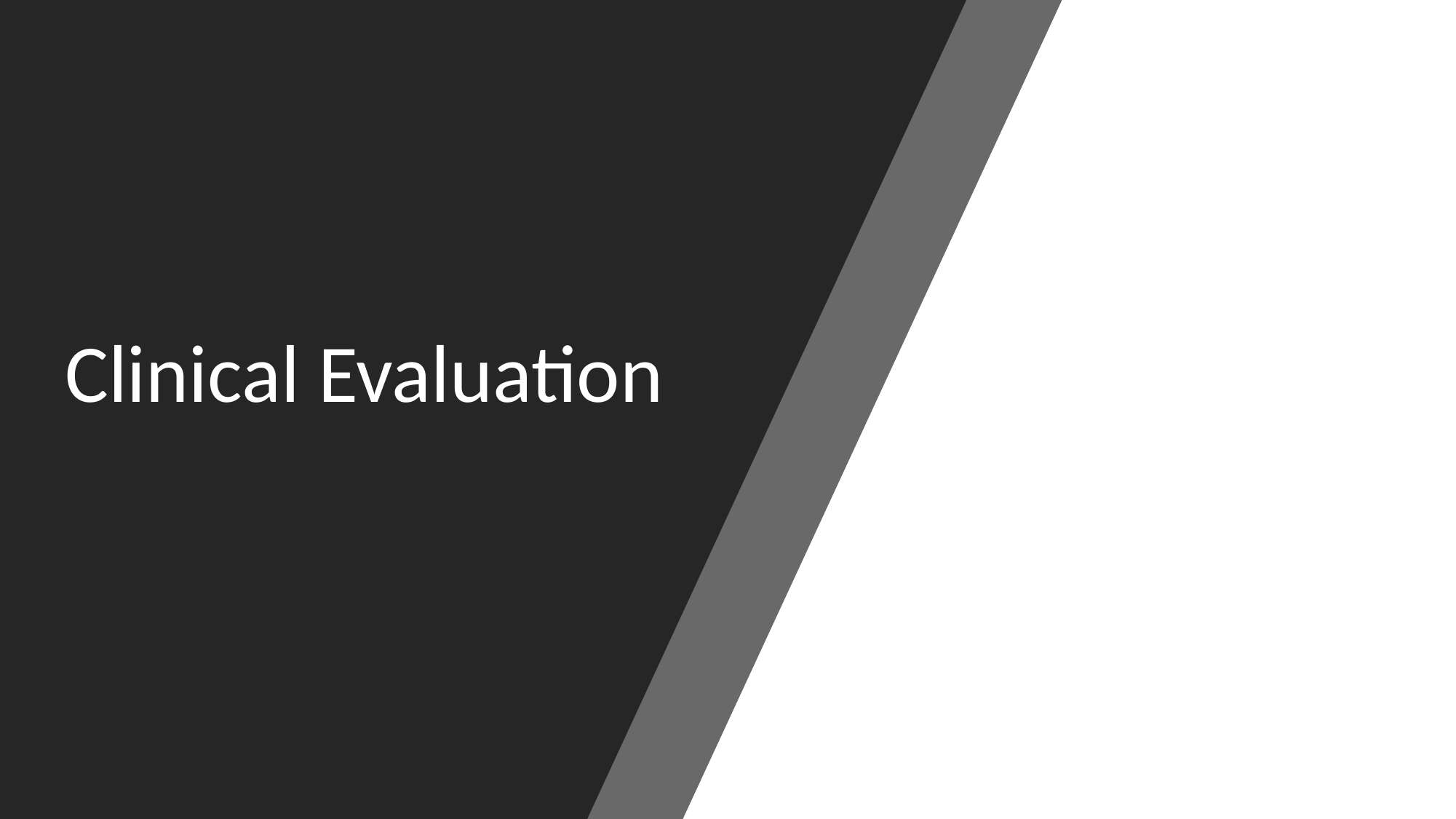

# Clinical Evaluation

## Slide 8
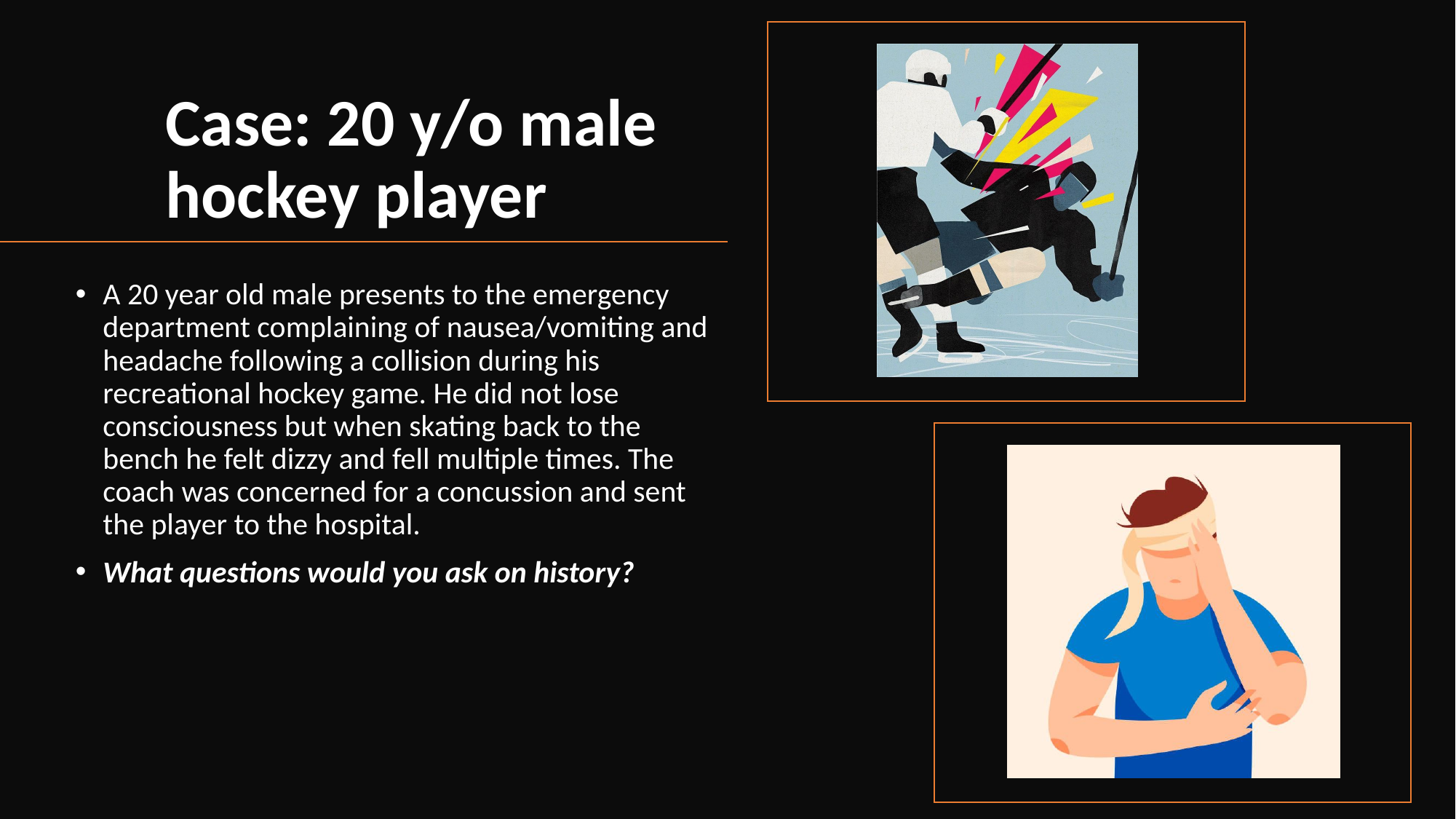

# Case: 20 y/o male hockey player
A 20 year old male presents to the emergency department complaining of nausea/vomiting and headache following a collision during his recreational hockey game. He did not lose consciousness but when skating back to the bench he felt dizzy and fell multiple times. The coach was concerned for a concussion and sent the player to the hospital.
What questions would you ask on history?

## Slide 9
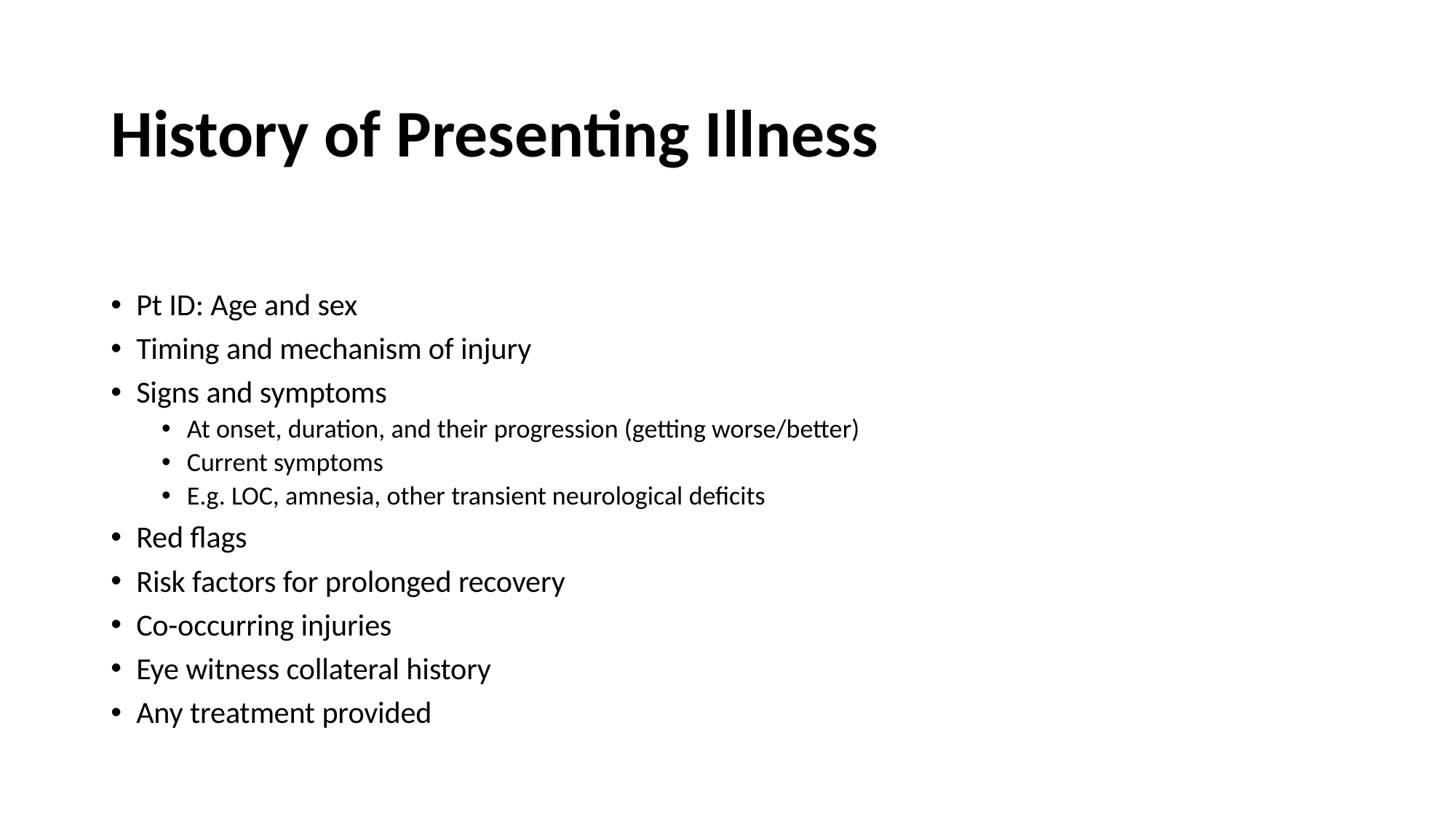

# History of Presenting Illness
Pt ID: Age and sex
Timing and mechanism of injury
Signs and symptoms
At onset, duration, and their progression (getting worse/better)
Current symptoms
E.g. LOC, amnesia, other transient neurological deficits
Red flags
Risk factors for prolonged recovery
Co-occurring injuries
Eye witness collateral history
Any treatment provided

## Slide 10
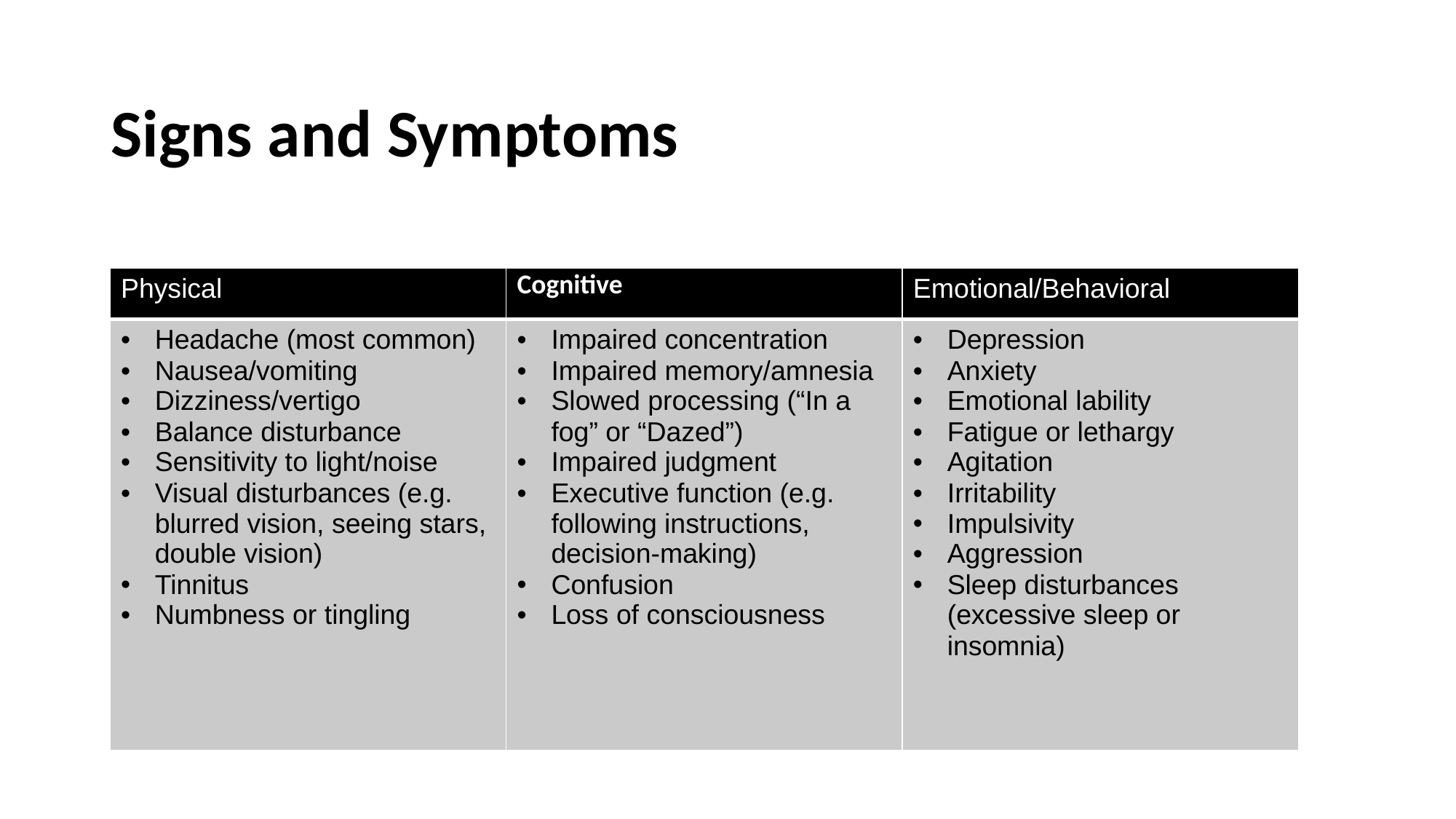

# Signs and Symptoms
| Physical | Cognitive | Emotional/Behavioral |
| --- | --- | --- |
| Headache (most common) Nausea/vomiting Dizziness/vertigo Balance disturbance Sensitivity to light/noise Visual disturbances (e.g. blurred vision, seeing stars, double vision) Tinnitus Numbness or tingling | Impaired concentration Impaired memory/amnesia Slowed processing (“In a fog” or “Dazed”) Impaired judgment Executive function (e.g. following instructions, decision-making) Confusion Loss of consciousness | Depression Anxiety Emotional lability Fatigue or lethargy Agitation Irritability Impulsivity Aggression Sleep disturbances (excessive sleep or insomnia) |

## Slide 11
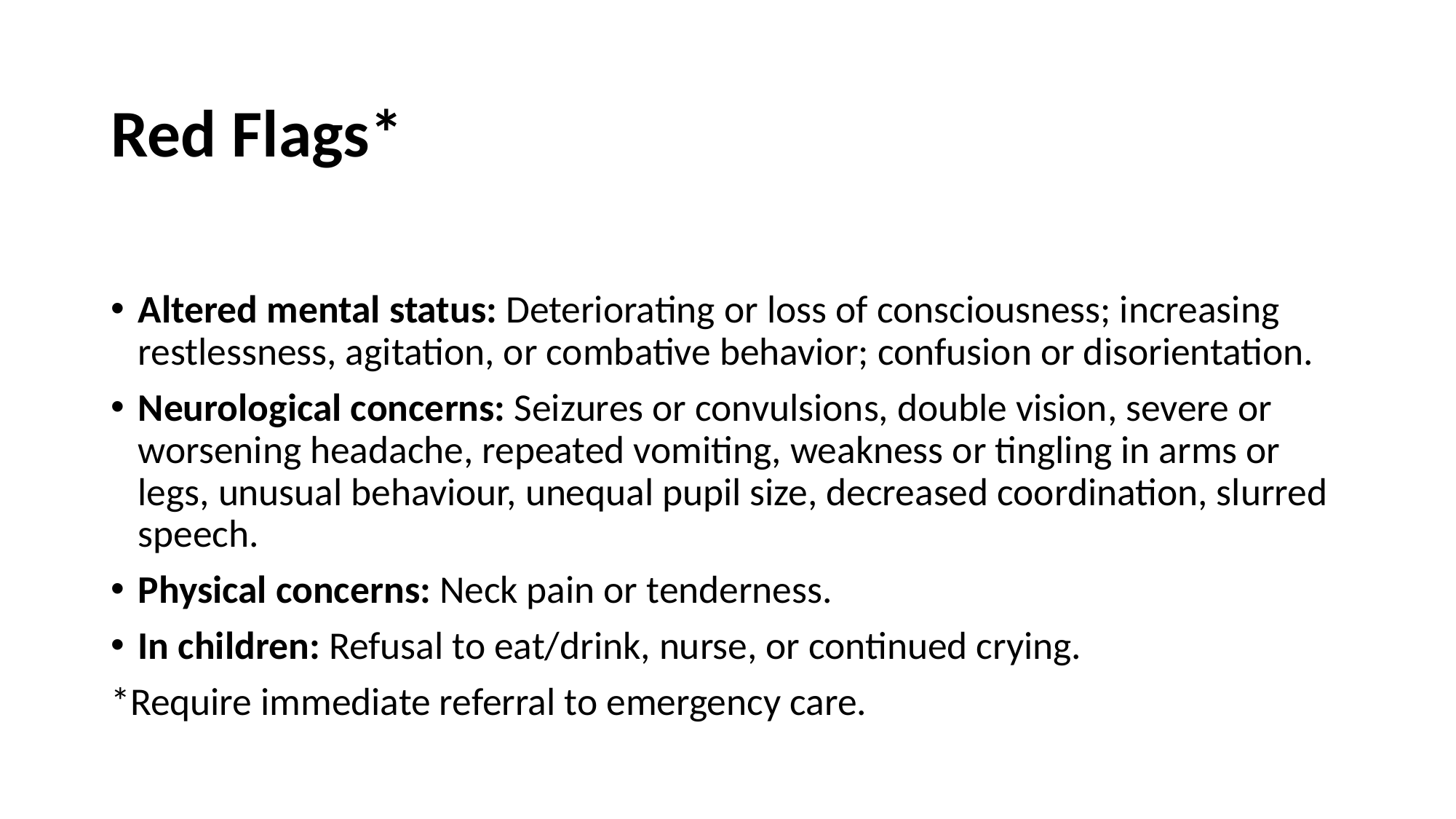

# Red Flags*
Altered mental status: Deteriorating or loss of consciousness; increasing restlessness, agitation, or combative behavior; confusion or disorientation.
Neurological concerns: Seizures or convulsions, double vision, severe or worsening headache, repeated vomiting, weakness or tingling in arms or legs, unusual behaviour, unequal pupil size, decreased coordination, slurred speech.
Physical concerns: Neck pain or tenderness.
In children: Refusal to eat/drink, nurse, or continued crying.
*Require immediate referral to emergency care.

## Slide 12
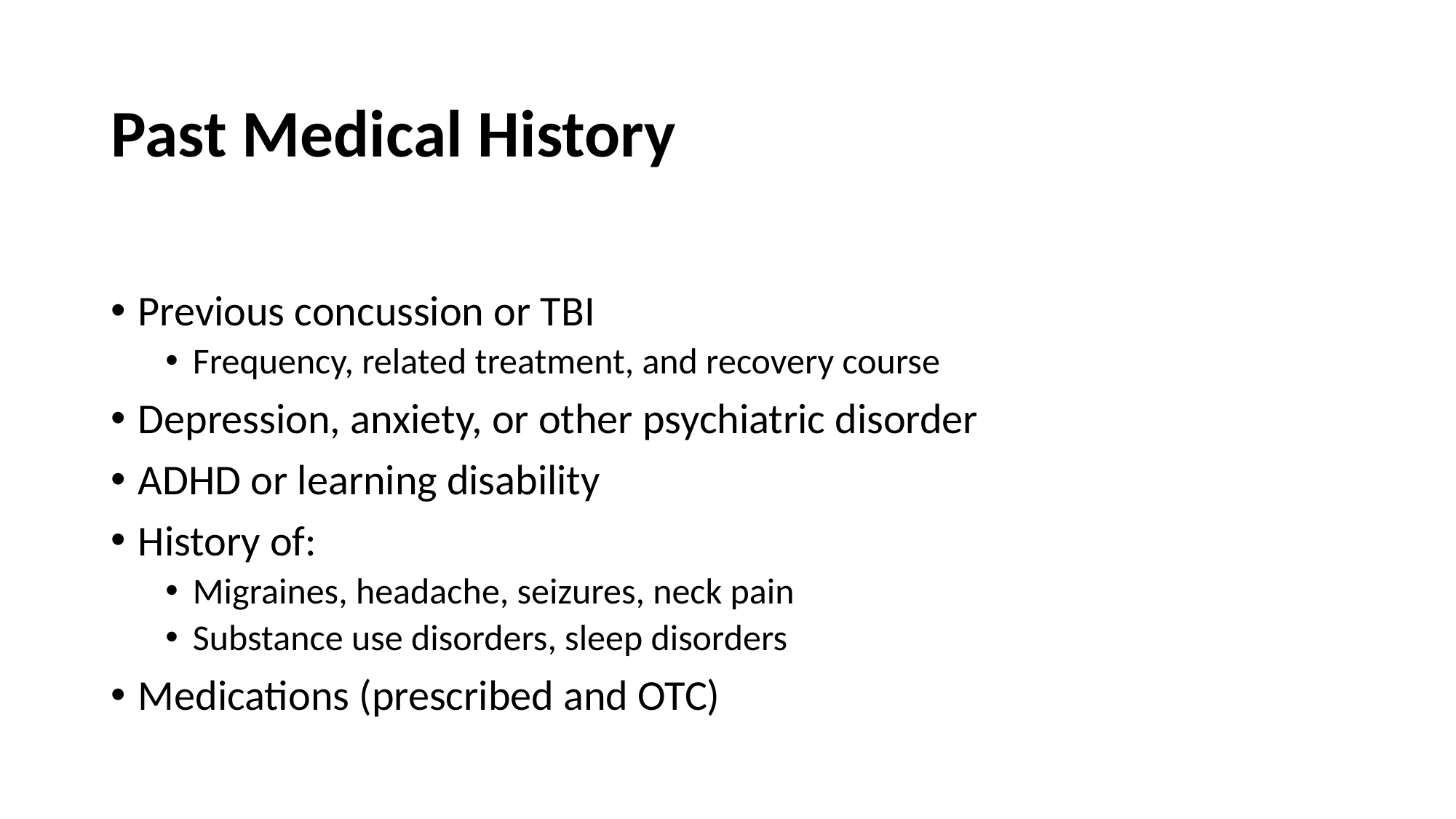

# Past Medical History
Previous concussion or TBI
Frequency, related treatment, and recovery course
Depression, anxiety, or other psychiatric disorder
ADHD or learning disability
History of:
Migraines, headache, seizures, neck pain
Substance use disorders, sleep disorders
Medications (prescribed and OTC)

## Slide 13
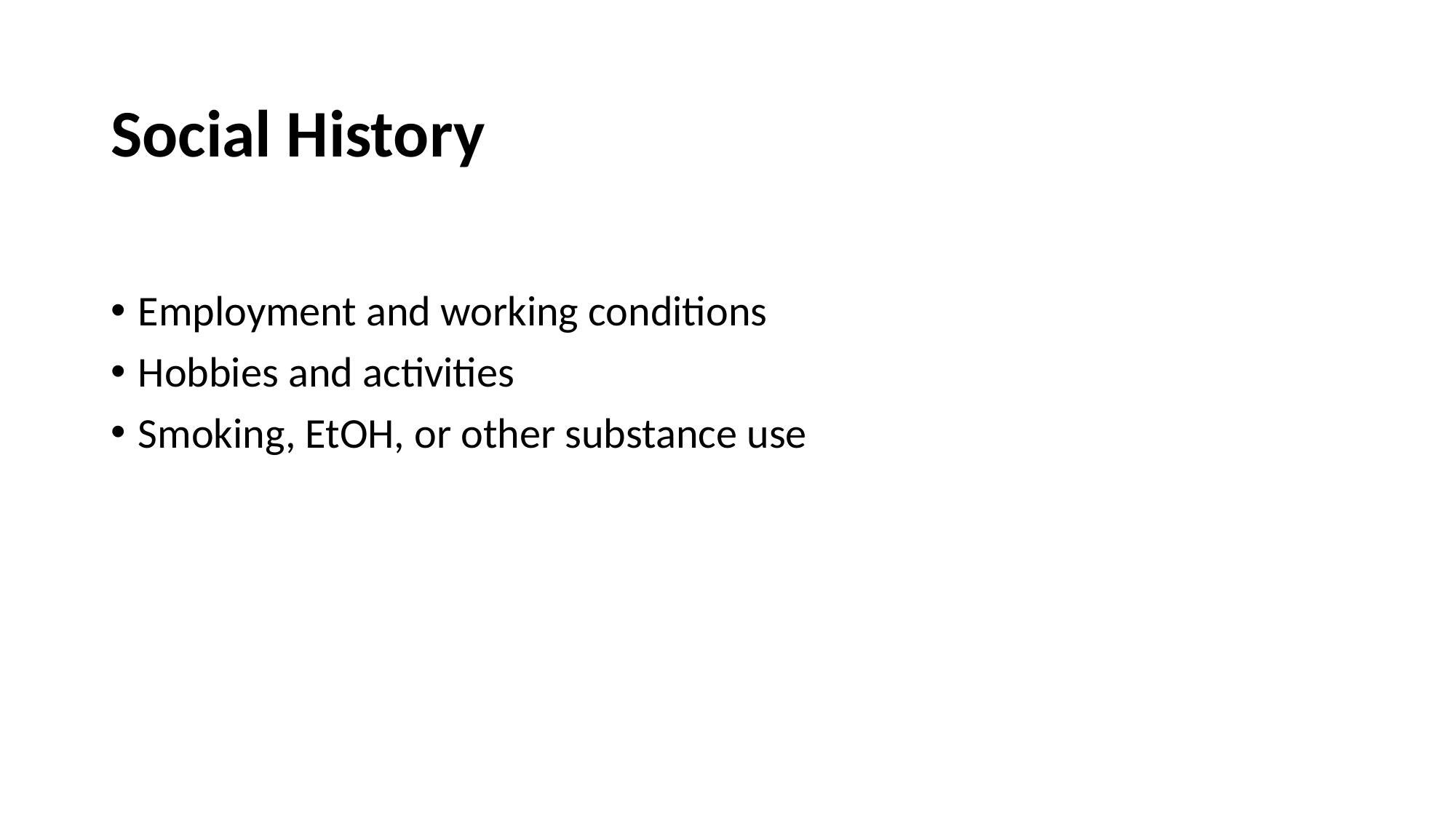

# Social History
Employment and working conditions
Hobbies and activities
Smoking, EtOH, or other substance use

## Slide 14
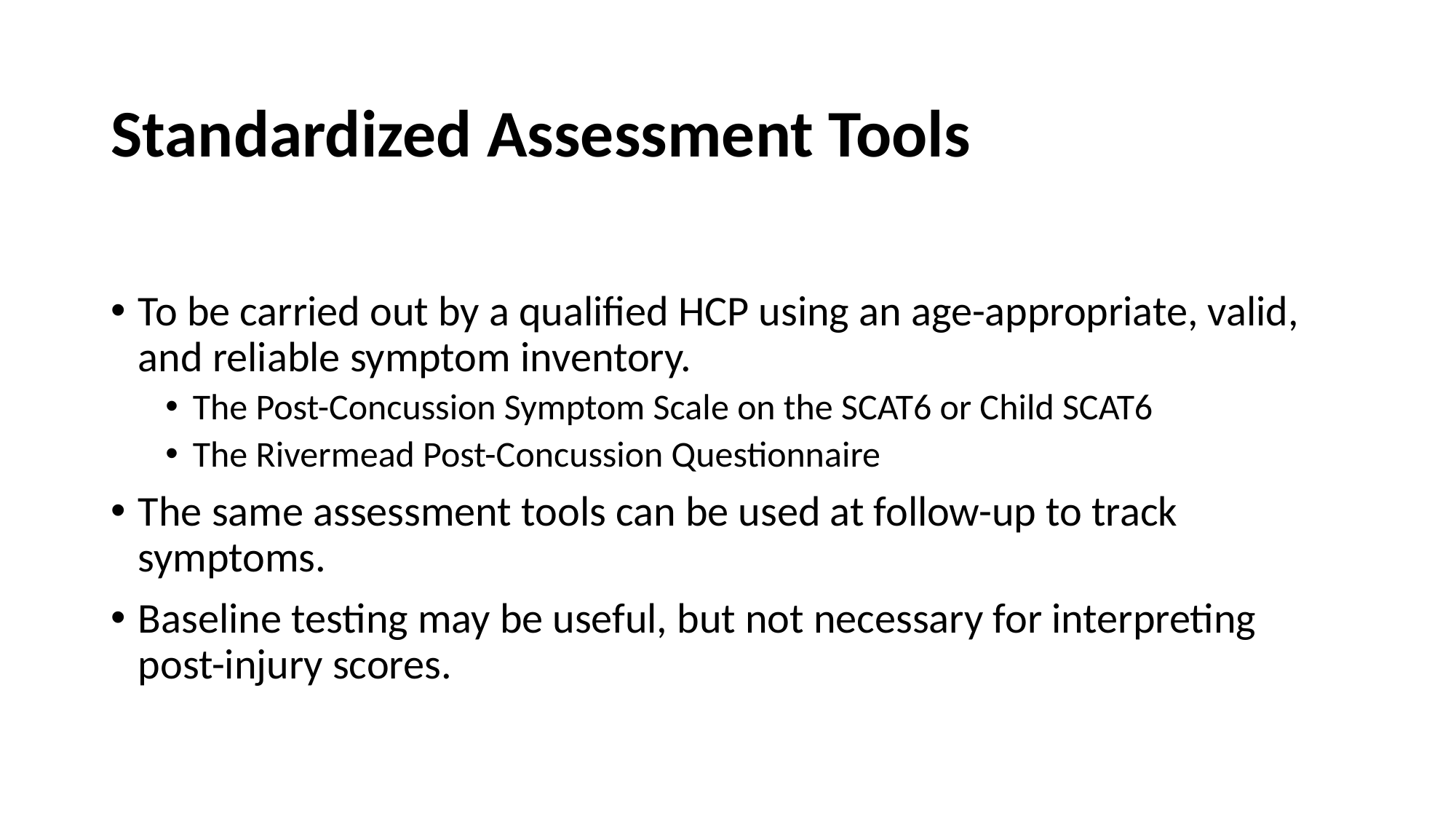

# Standardized Assessment Tools
To be carried out by a qualified HCP using an age-appropriate, valid, and reliable symptom inventory.
The Post-Concussion Symptom Scale on the SCAT6 or Child SCAT6
The Rivermead Post-Concussion Questionnaire
The same assessment tools can be used at follow-up to track symptoms.
Baseline testing may be useful, but not necessary for interpreting post-injury scores.

## Slide 15
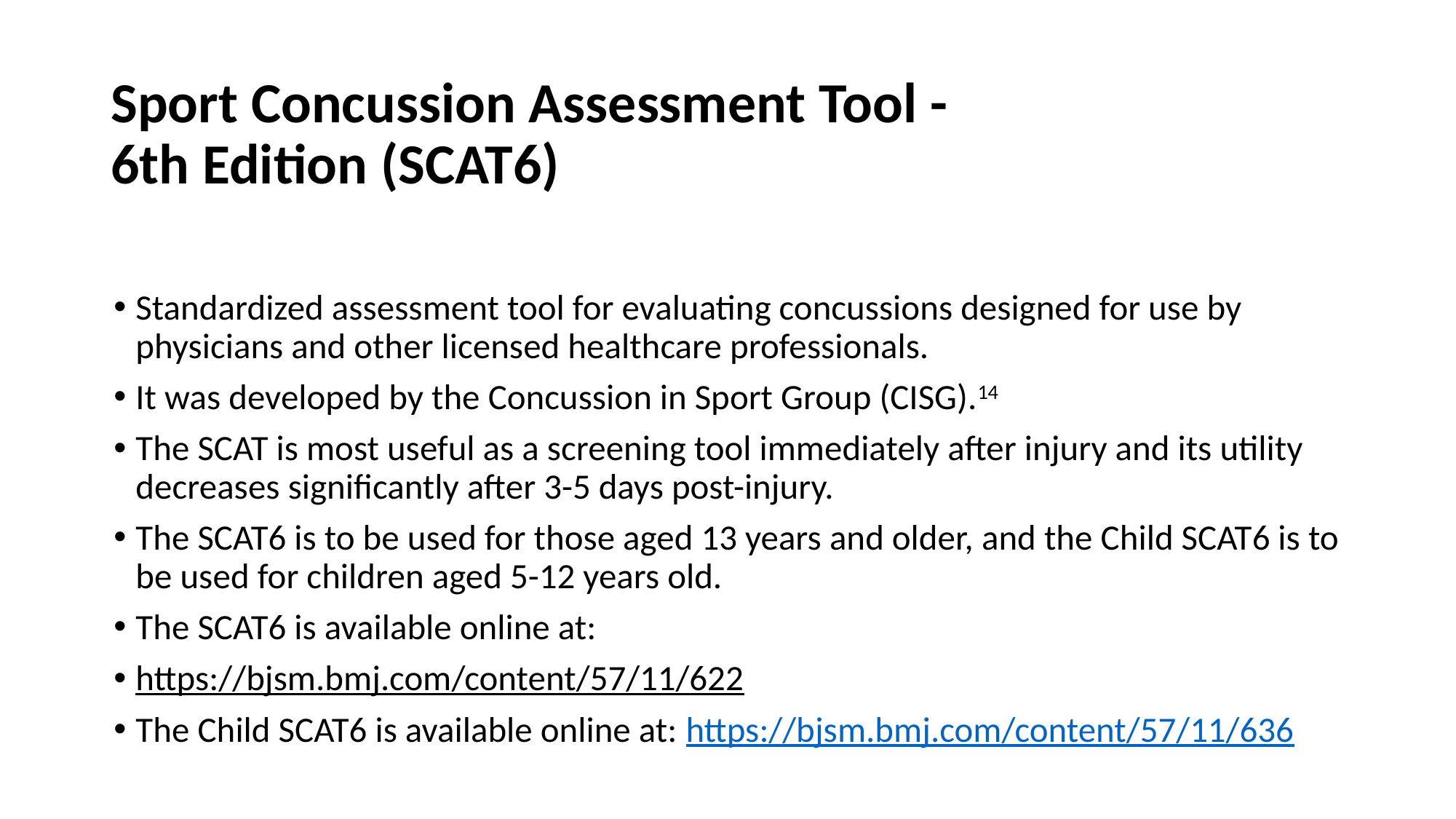

# Sport Concussion Assessment Tool - 6th Edition (SCAT6)
Standardized assessment tool for evaluating concussions designed for use by physicians and other licensed healthcare professionals.
It was developed by the Concussion in Sport Group (CISG).14
The SCAT is most useful as a screening tool immediately after injury and its utility decreases significantly after 3-5 days post-injury.
The SCAT6 is to be used for those aged 13 years and older, and the Child SCAT6 is to be used for children aged 5-12 years old.
The SCAT6 is available online at:
https://bjsm.bmj.com/content/57/11/622
The Child SCAT6 is available online at: https://bjsm.bmj.com/content/57/11/636

## Slide 16
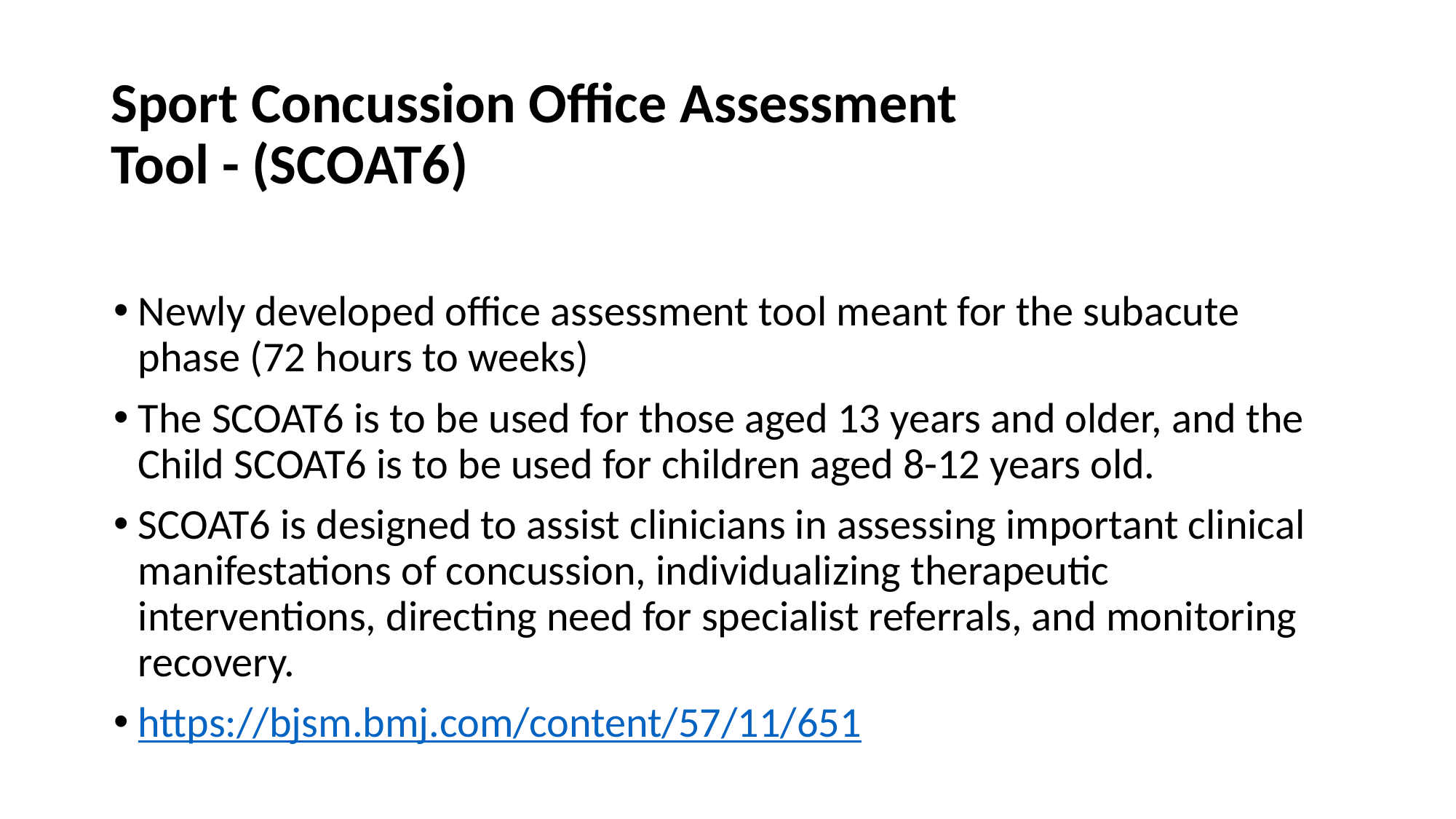

# Sport Concussion Office Assessment Tool - (SCOAT6)
Newly developed office assessment tool meant for the subacute phase (72 hours to weeks)
The SCOAT6 is to be used for those aged 13 years and older, and the Child SCOAT6 is to be used for children aged 8-12 years old.
SCOAT6 is designed to assist clinicians in assessing important clinical manifestations of concussion, individualizing therapeutic interventions, directing need for specialist referrals, and monitoring recovery.
https://bjsm.bmj.com/content/57/11/651

## Slide 17
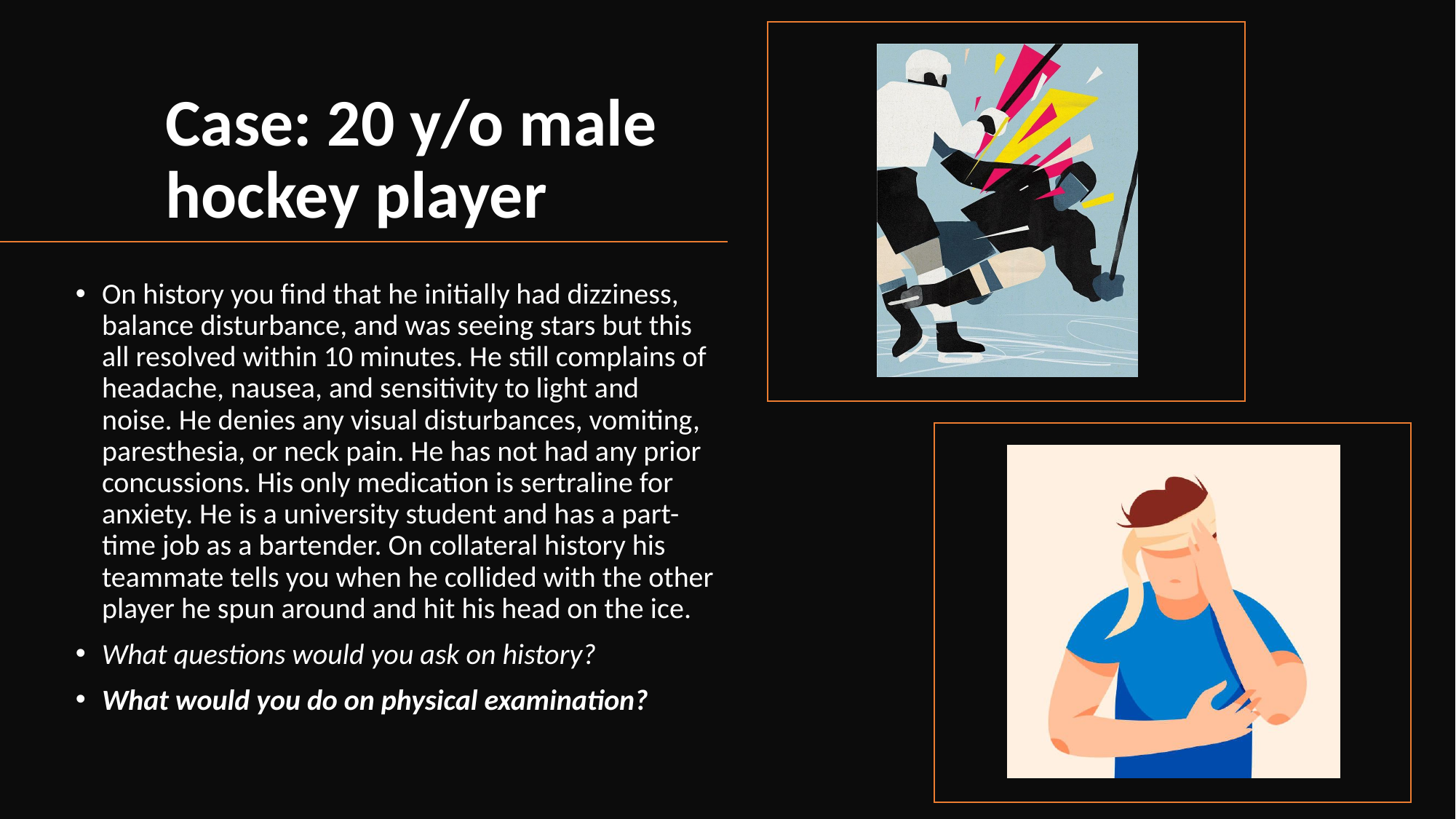

# Case: 20 y/o male hockey player
On history you find that he initially had dizziness, balance disturbance, and was seeing stars but this all resolved within 10 minutes. He still complains of headache, nausea, and sensitivity to light and noise. He denies any visual disturbances, vomiting, paresthesia, or neck pain. He has not had any prior concussions. His only medication is sertraline for anxiety. He is a university student and has a part-time job as a bartender. On collateral history his teammate tells you when he collided with the other player he spun around and hit his head on the ice.
What questions would you ask on history?
What would you do on physical examination?

## Slide 18
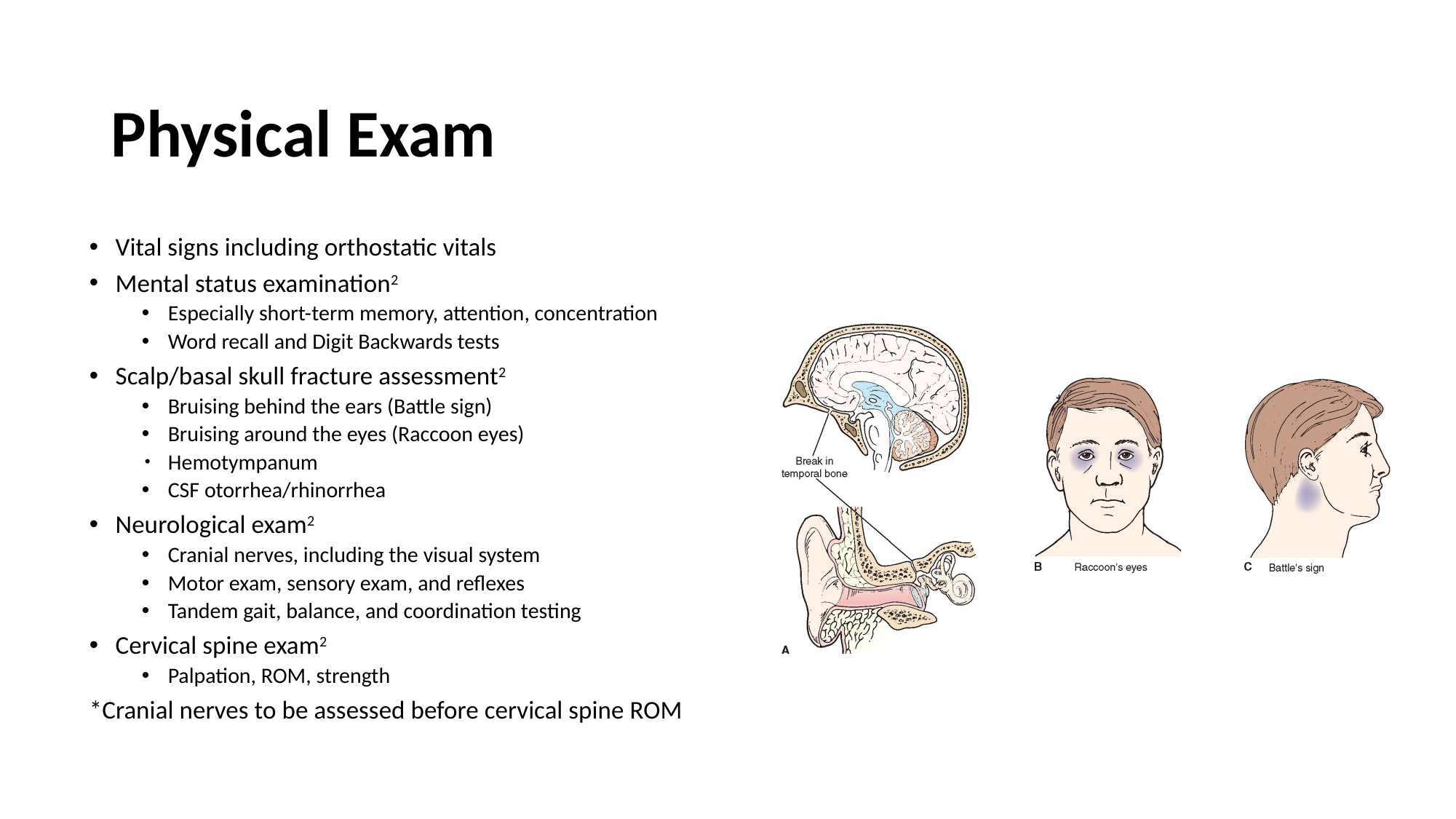

# Physical Exam
Vital signs including orthostatic vitals
Mental status examination2
Especially short-term memory, attention, concentration
Word recall and Digit Backwards tests
Scalp/basal skull fracture assessment2
Bruising behind the ears (Battle sign)
Bruising around the eyes (Raccoon eyes)
Hemotympanum
CSF otorrhea/rhinorrhea
Neurological exam2
Cranial nerves, including the visual system
Motor exam, sensory exam, and reflexes
Tandem gait, balance, and coordination testing
Cervical spine exam2
Palpation, ROM, strength
*Cranial nerves to be assessed before cervical spine ROM

## Slide 19
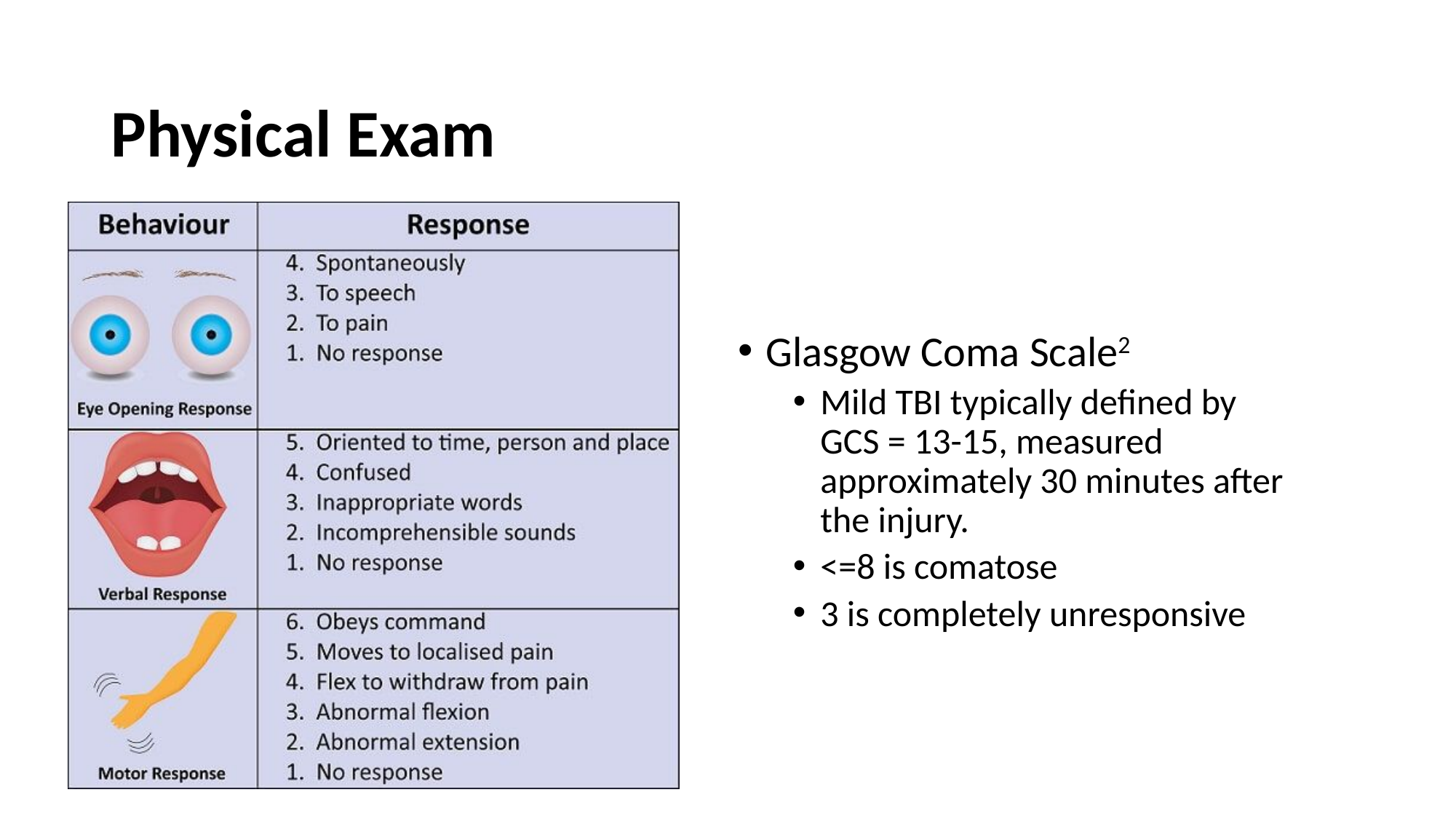

# Physical Exam
Glasgow Coma Scale2
Mild TBI typically defined by GCS = 13-15, measured approximately 30 minutes after the injury.
<=8 is comatose
3 is completely unresponsive

## Slide 20
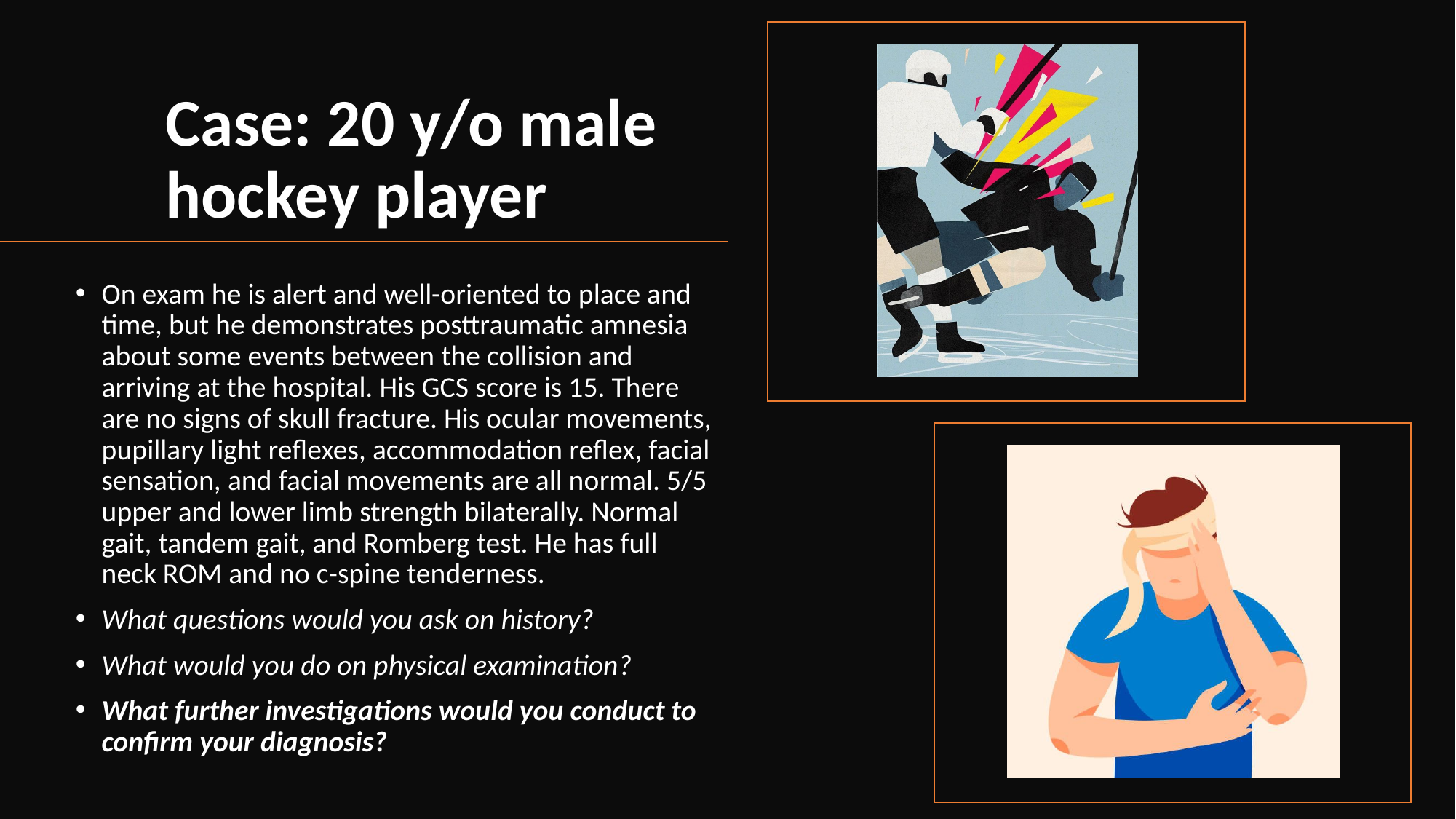

# Case: 20 y/o male hockey player
On exam he is alert and well-oriented to place and time, but he demonstrates posttraumatic amnesia about some events between the collision and arriving at the hospital. His GCS score is 15. There are no signs of skull fracture. His ocular movements, pupillary light reflexes, accommodation reflex, facial sensation, and facial movements are all normal. 5/5 upper and lower limb strength bilaterally. Normal gait, tandem gait, and Romberg test. He has full neck ROM and no c-spine tenderness.
What questions would you ask on history?
What would you do on physical examination?
What further investigations would you conduct to confirm your diagnosis?

## Slide 21
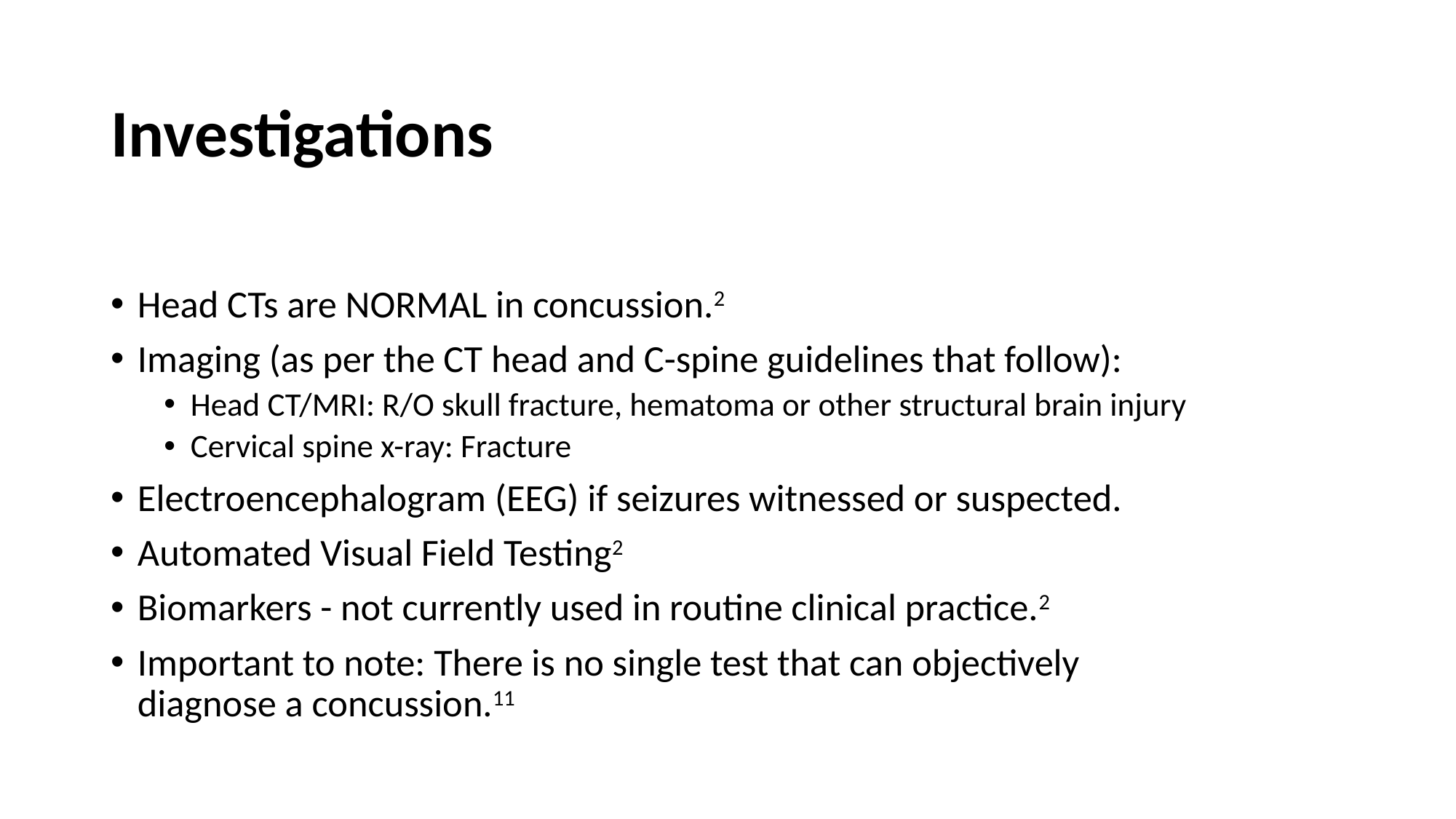

# Investigations
Head CTs are NORMAL in concussion.2
Imaging (as per the CT head and C-spine guidelines that follow):
Head CT/MRI: R/O skull fracture, hematoma or other structural brain injury
Cervical spine x-ray: Fracture
Electroencephalogram (EEG) if seizures witnessed or suspected.
Automated Visual Field Testing2
Biomarkers - not currently used in routine clinical practice.2
Important to note: There is no single test that can objectively diagnose a concussion.11

## Slide 22
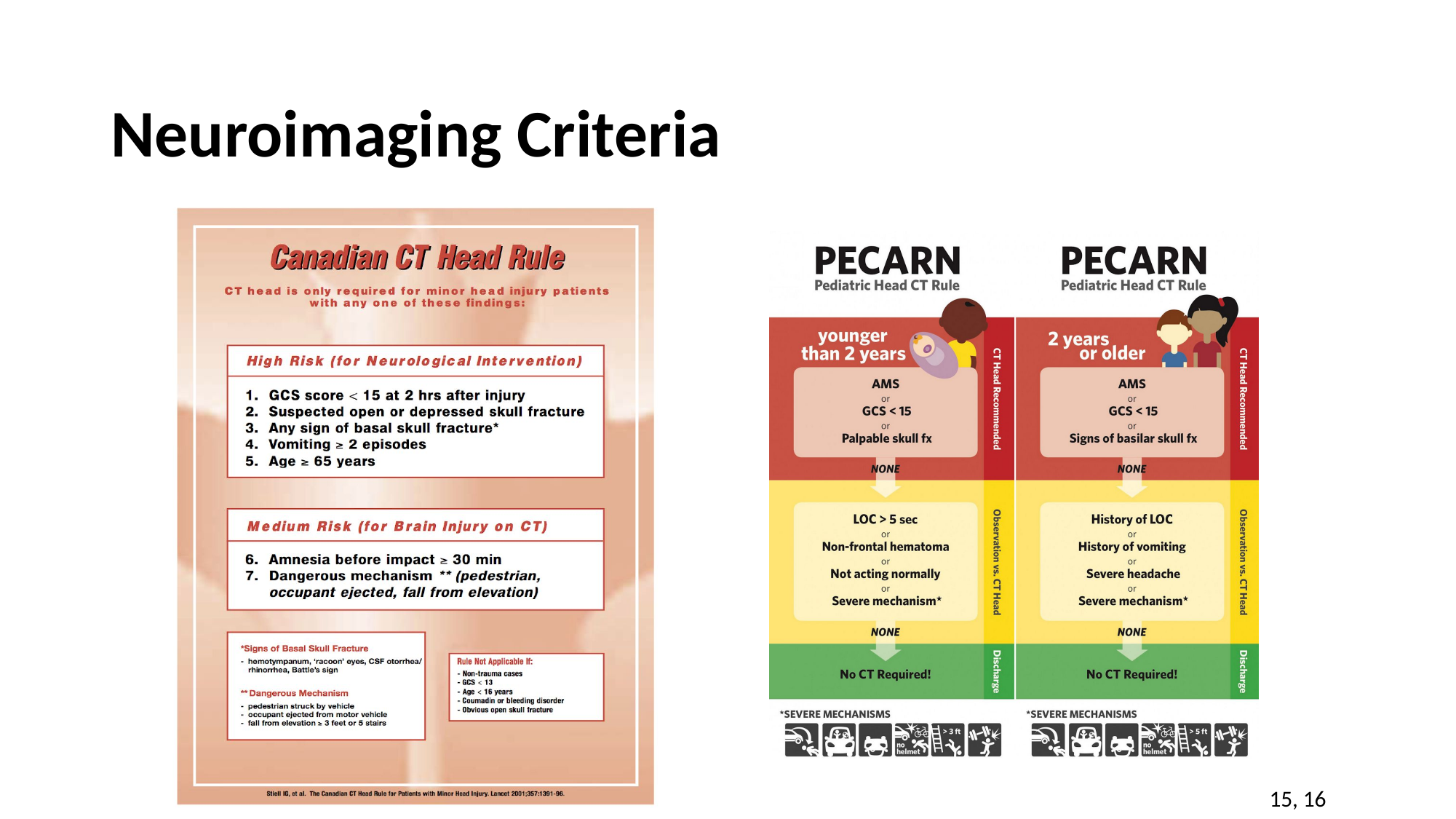

# Neuroimaging Criteria
15, 16

## Slide 23
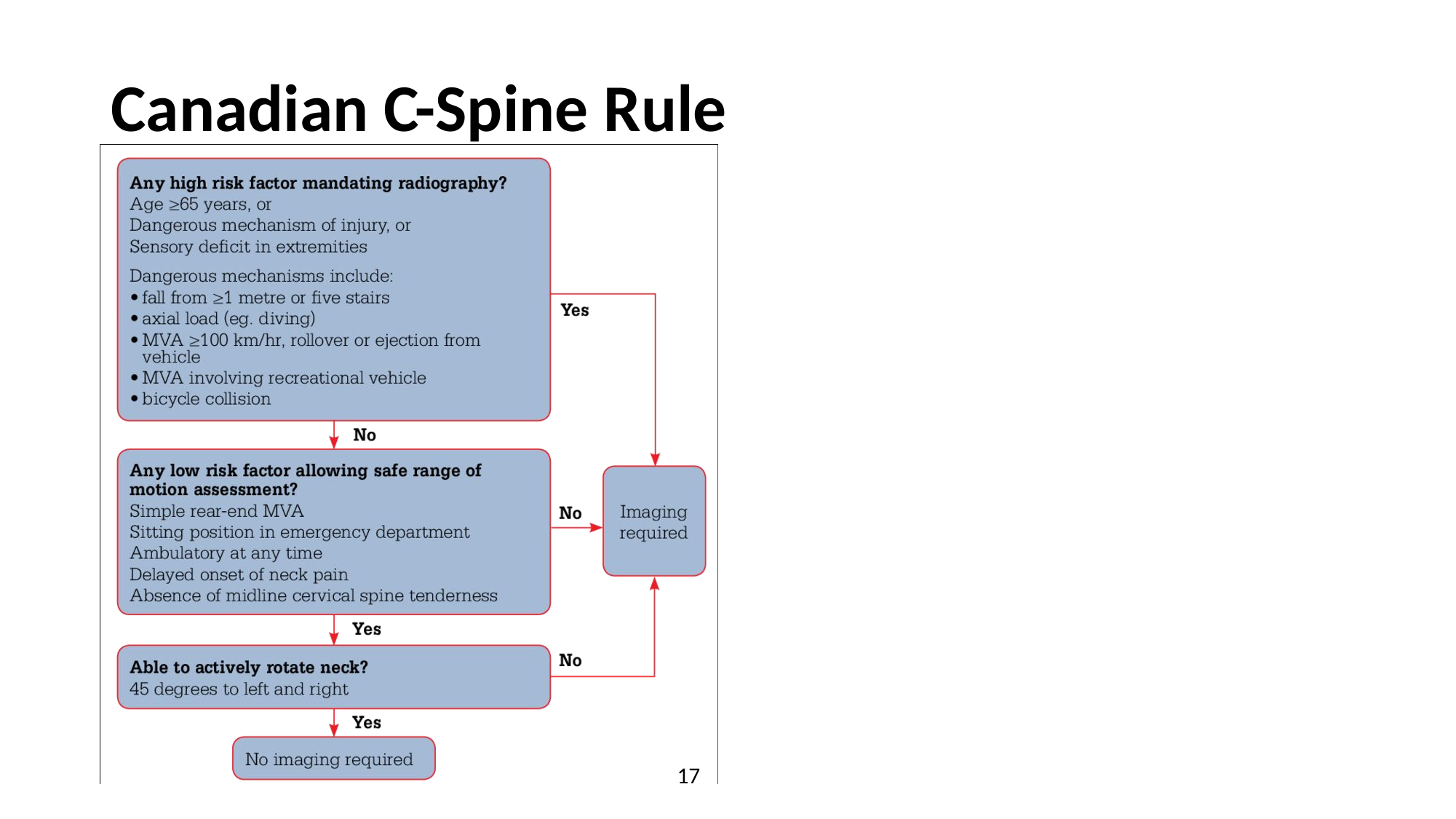

# Canadian C-Spine Rule
17

## Slide 24
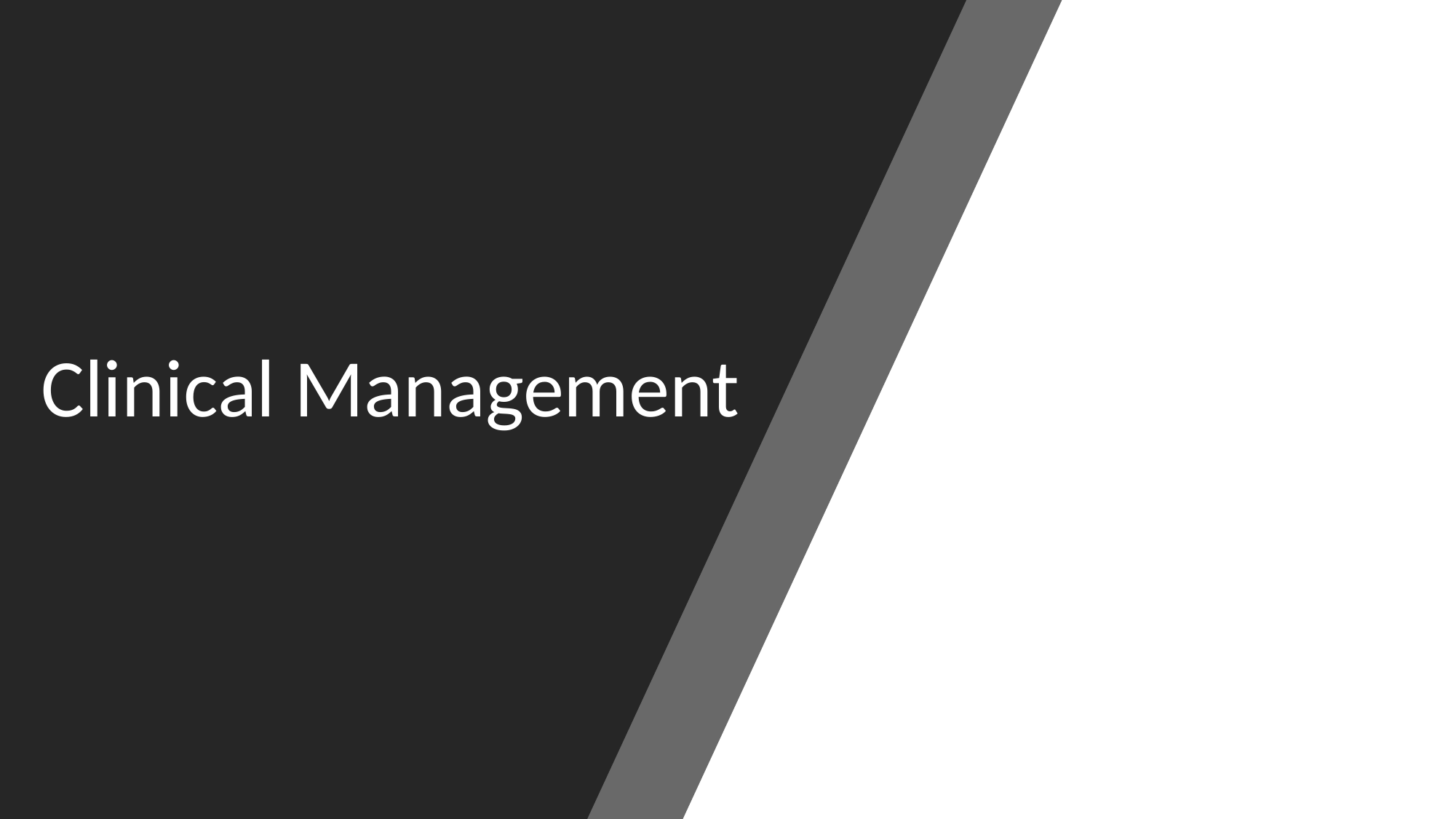

# Clinical Management

## Slide 25
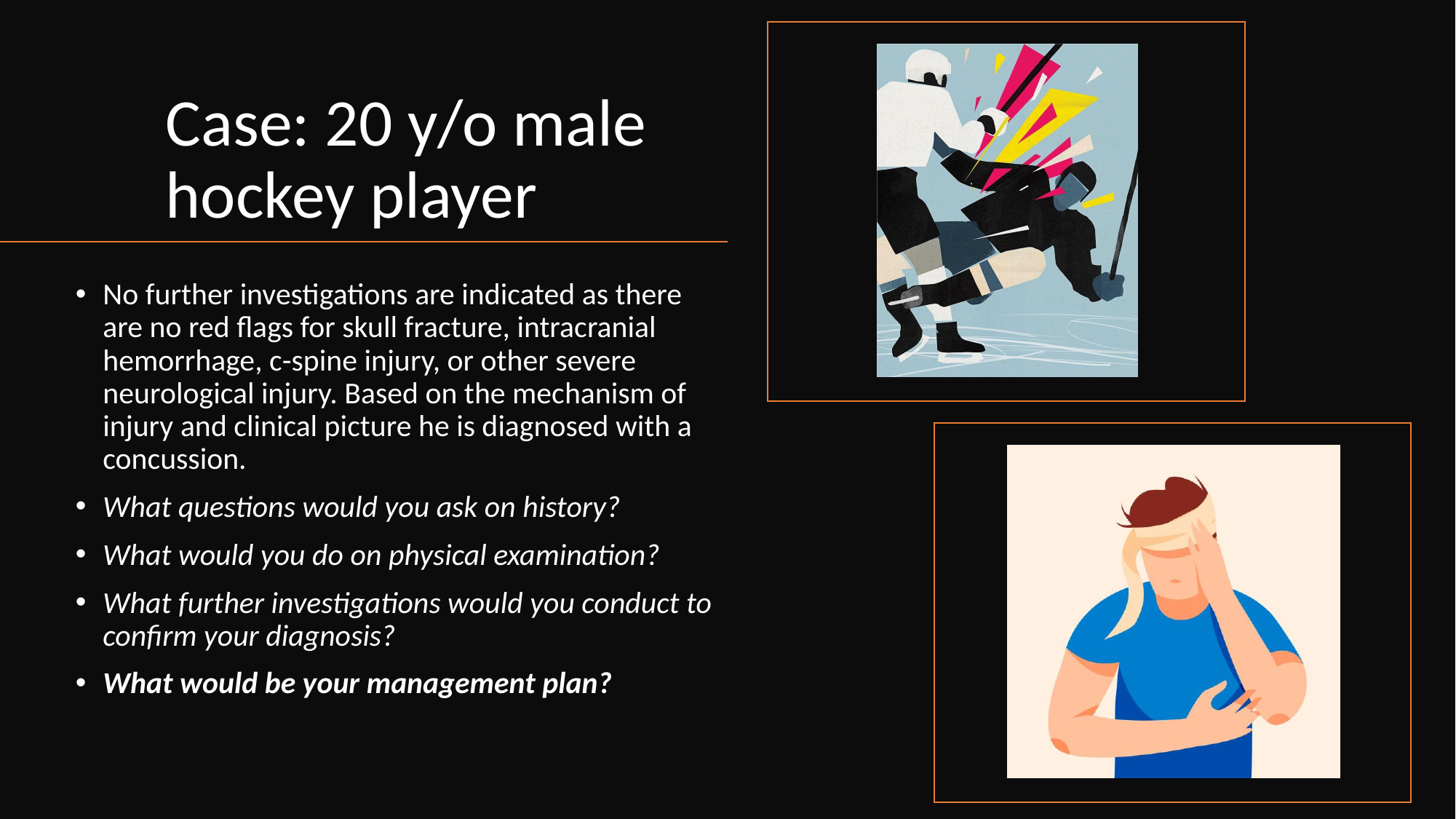

# Case: 20 y/o male hockey player
No further investigations are indicated as there are no red flags for skull fracture, intracranial hemorrhage, c-spine injury, or other severe neurological injury. Based on the mechanism of injury and clinical picture he is diagnosed with a concussion.
What questions would you ask on history?
What would you do on physical examination?
What further investigations would you conduct to confirm your diagnosis?
What would be your management plan?

## Slide 26
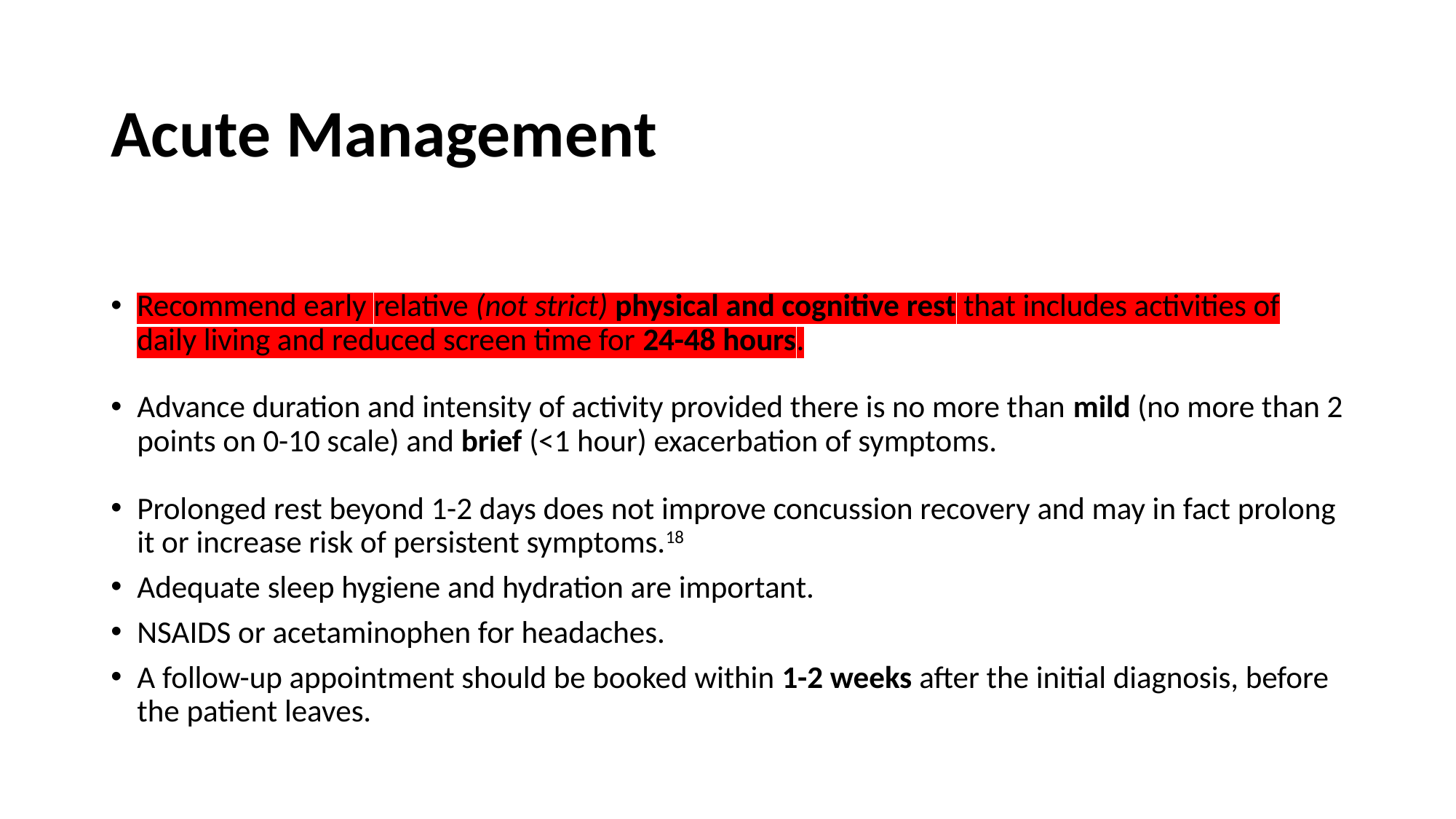

# Acute Management
Recommend early relative (not strict) physical and cognitive rest that includes activities of daily living and reduced screen time for 24-48 hours.
Advance duration and intensity of activity provided there is no more than mild (no more than 2 points on 0-10 scale) and brief (<1 hour) exacerbation of symptoms.
Prolonged rest beyond 1-2 days does not improve concussion recovery and may in fact prolong it or increase risk of persistent symptoms.18
Adequate sleep hygiene and hydration are important.
NSAIDS or acetaminophen for headaches.
A follow-up appointment should be booked within 1-2 weeks after the initial diagnosis, before the patient leaves.

## Slide 27
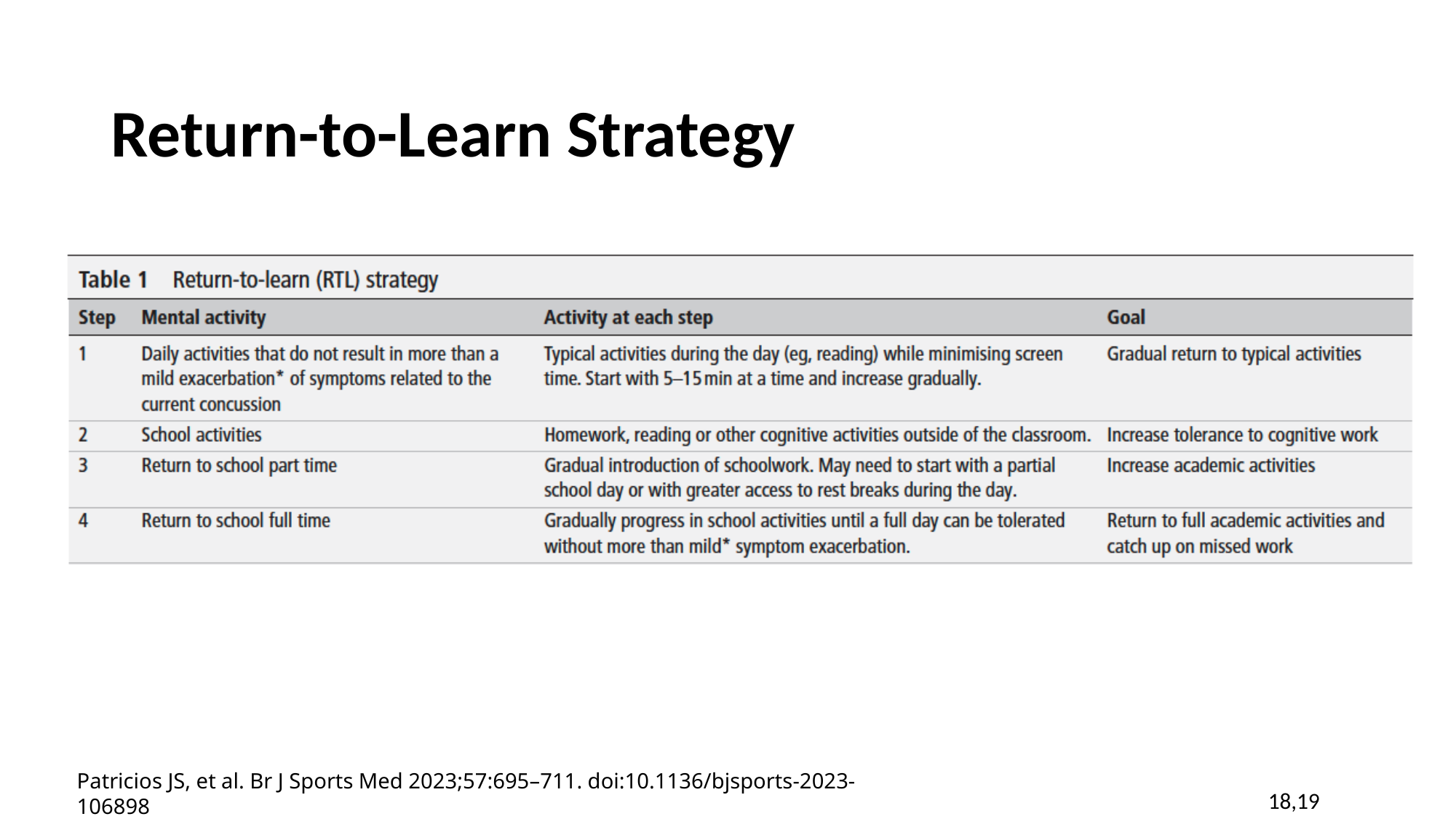

# Return-to-Learn Strategy
Patricios JS, et al. Br J Sports Med 2023;57:695–711. doi:10.1136/bjsports-2023-106898
18,19

## Slide 28
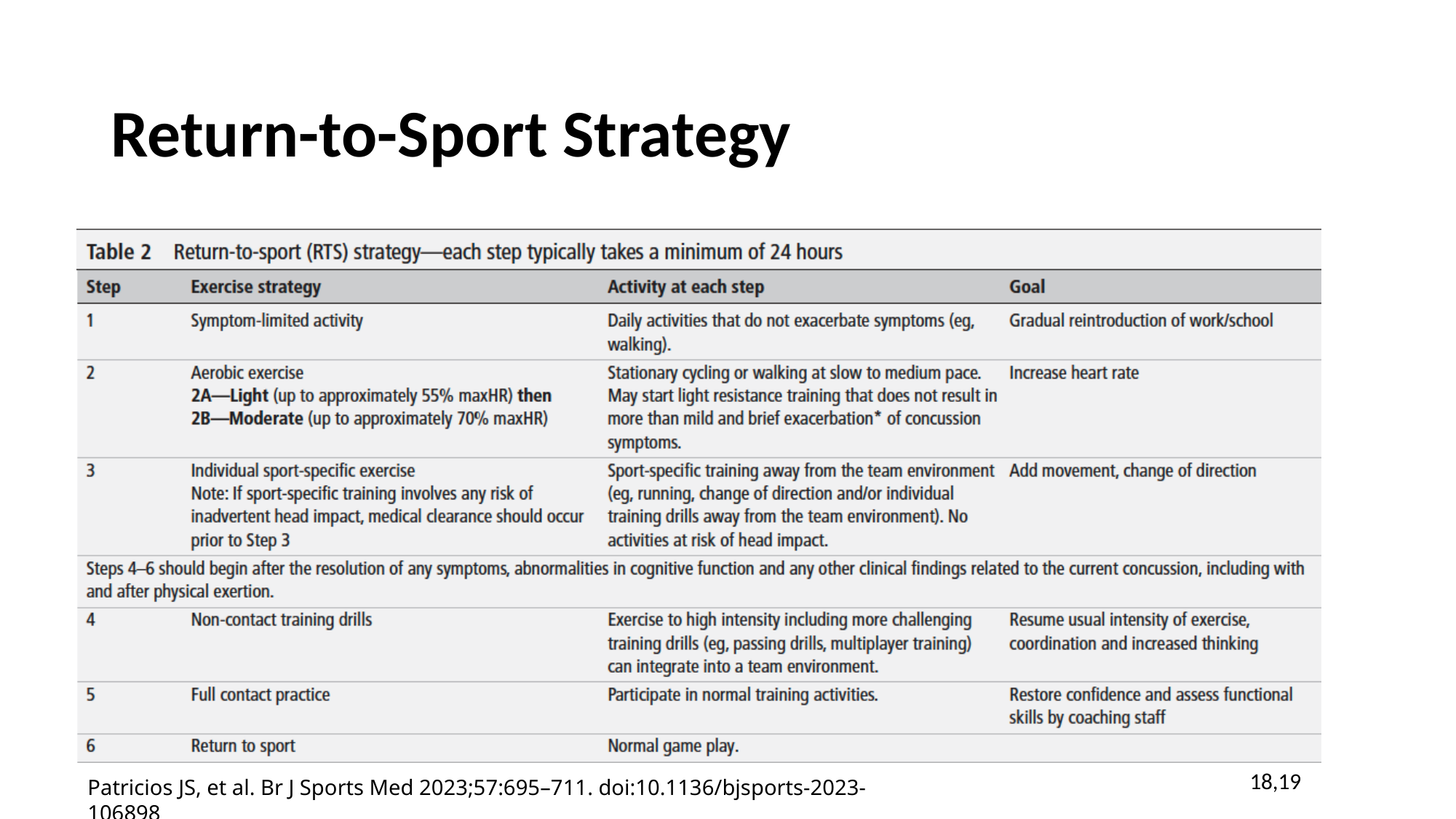

# Return-to-Sport Strategy
18,19
Patricios JS, et al. Br J Sports Med 2023;57:695–711. doi:10.1136/bjsports-2023-106898

## Slide 29
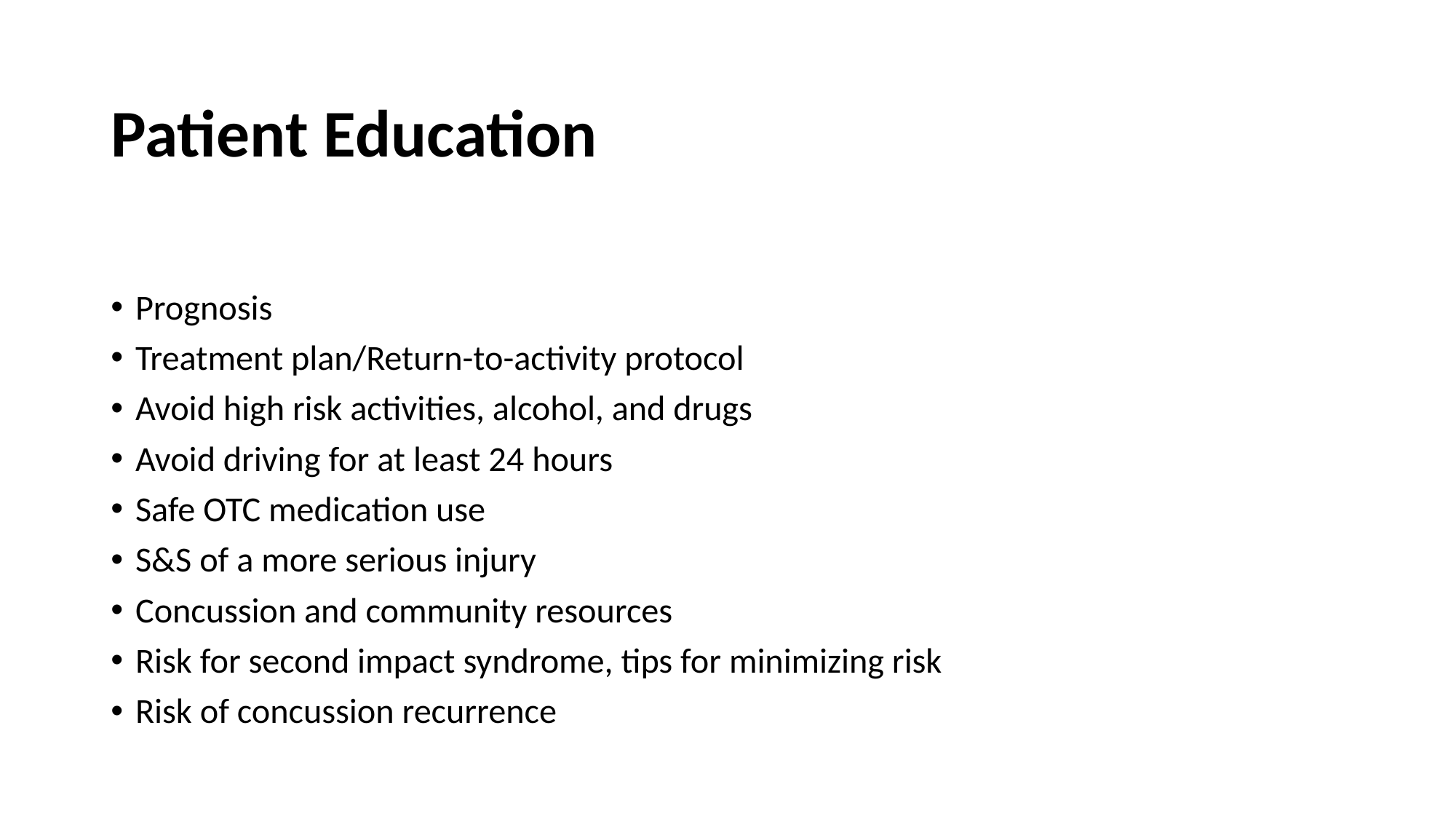

# Patient Education
Prognosis
Treatment plan/Return-to-activity protocol
Avoid high risk activities, alcohol, and drugs
Avoid driving for at least 24 hours
Safe OTC medication use
S&S of a more serious injury
Concussion and community resources
Risk for second impact syndrome, tips for minimizing risk
Risk of concussion recurrence

## Slide 30
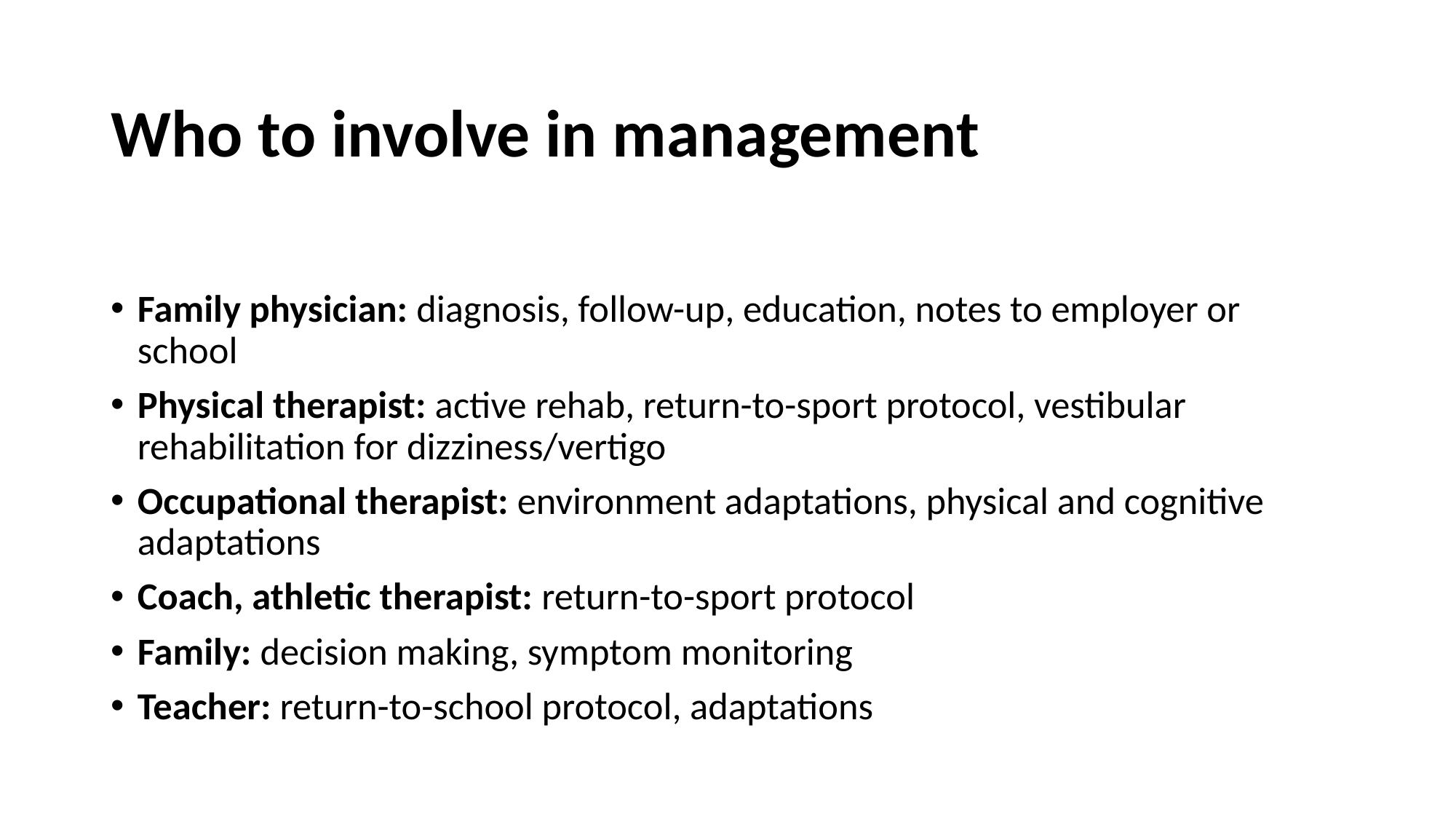

# Who to involve in management
Family physician: diagnosis, follow-up, education, notes to employer or school
Physical therapist: active rehab, return-to-sport protocol, vestibular rehabilitation for dizziness/vertigo
Occupational therapist: environment adaptations, physical and cognitive adaptations
Coach, athletic therapist: return-to-sport protocol
Family: decision making, symptom monitoring
Teacher: return-to-school protocol, adaptations

## Slide 31
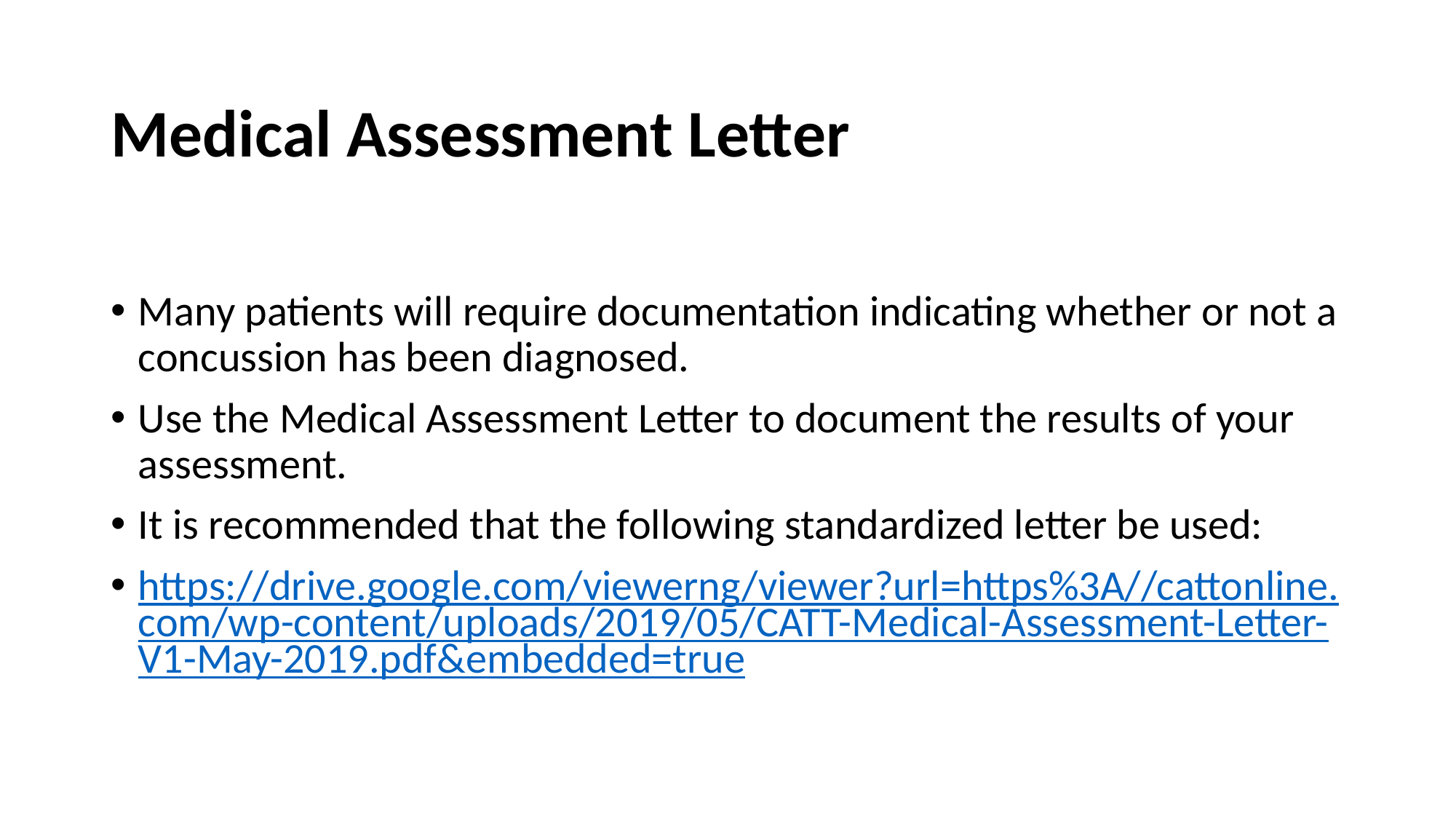

# Medical Assessment Letter
Many patients will require documentation indicating whether or not a concussion has been diagnosed.
Use the Medical Assessment Letter to document the results of your assessment.
It is recommended that the following standardized letter be used:
https://drive.google.com/viewerng/viewer?url=https%3A//cattonline.com/wp-content/uploads/2019/05/CATT-Medical-Assessment-Letter-V1-May-2019.pdf&embedded=true

## Slide 32
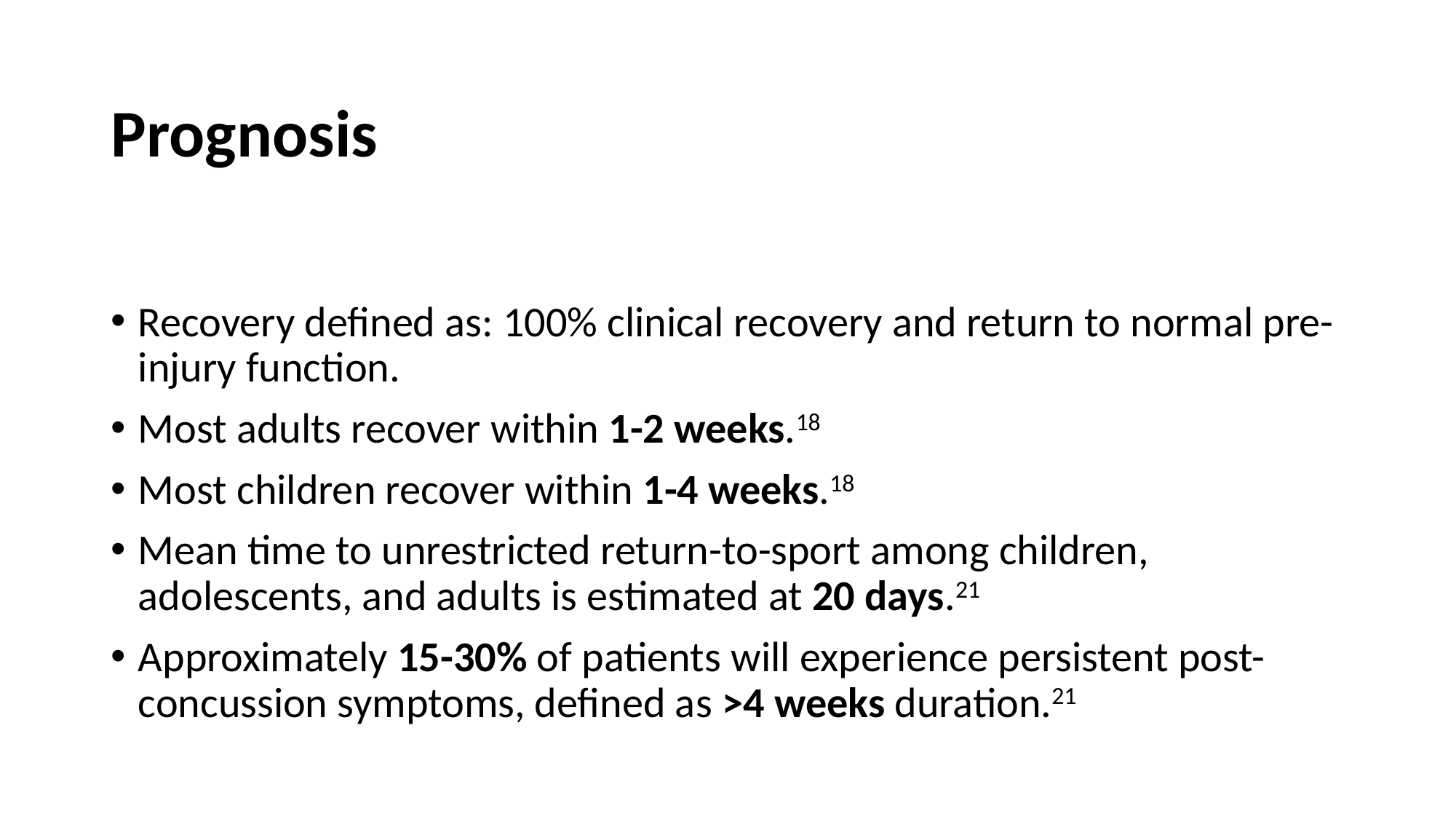

# Prognosis
Recovery defined as: 100% clinical recovery and return to normal pre-injury function.
Most adults recover within 1-2 weeks.18
Most children recover within 1-4 weeks.18
Mean time to unrestricted return-to-sport among children, adolescents, and adults is estimated at 20 days.21
Approximately 15-30% of patients will experience persistent post-concussion symptoms, defined as >4 weeks duration.21

## Slide 33
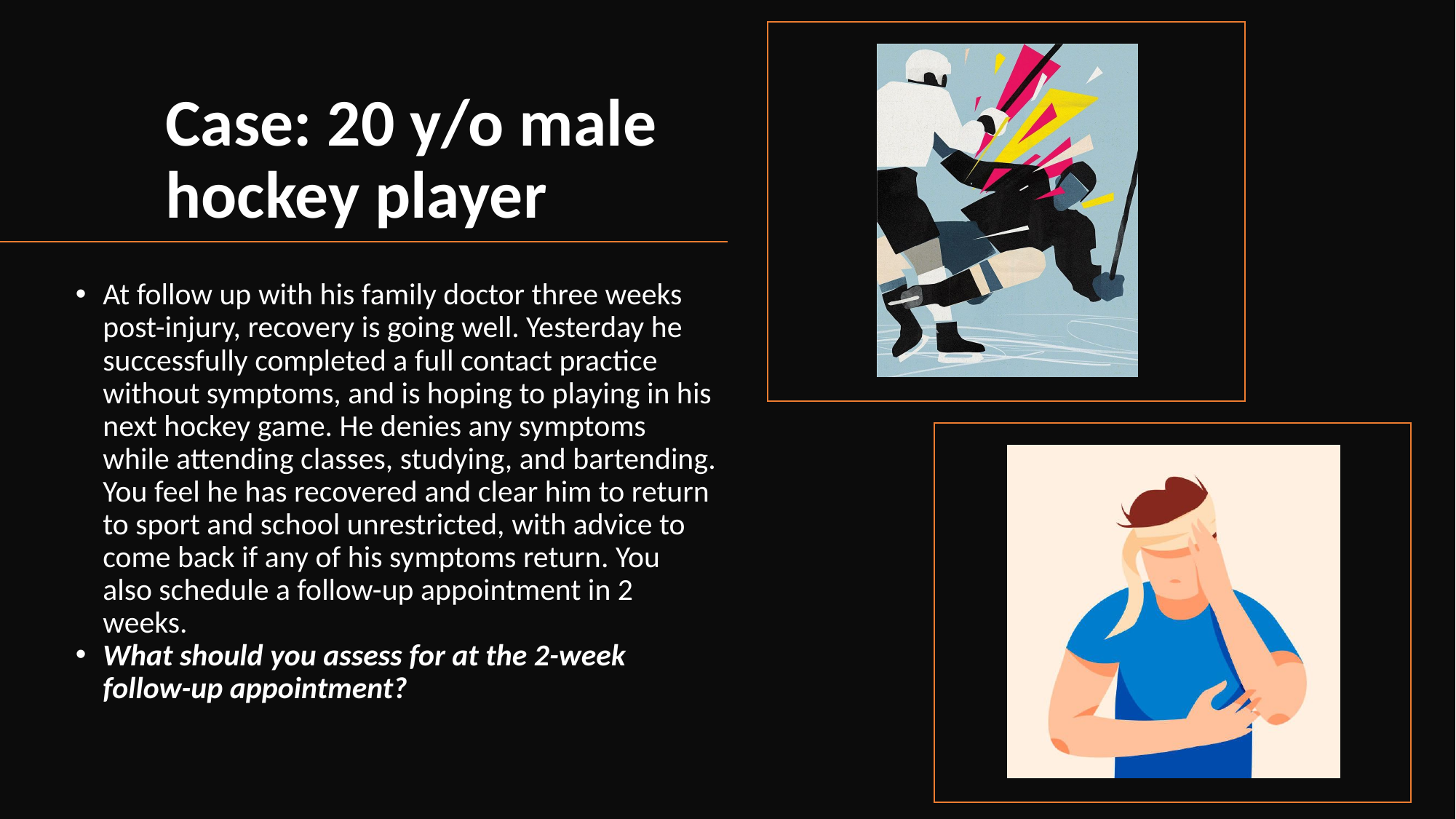

# Case: 20 y/o male hockey player
At follow up with his family doctor three weeks post-injury, recovery is going well. Yesterday he successfully completed a full contact practice without symptoms, and is hoping to playing in his next hockey game. He denies any symptoms while attending classes, studying, and bartending. You feel he has recovered and clear him to return to sport and school unrestricted, with advice to come back if any of his symptoms return. You also schedule a follow-up appointment in 2 weeks.
What should you assess for at the 2-week follow-up appointment?

## Slide 34
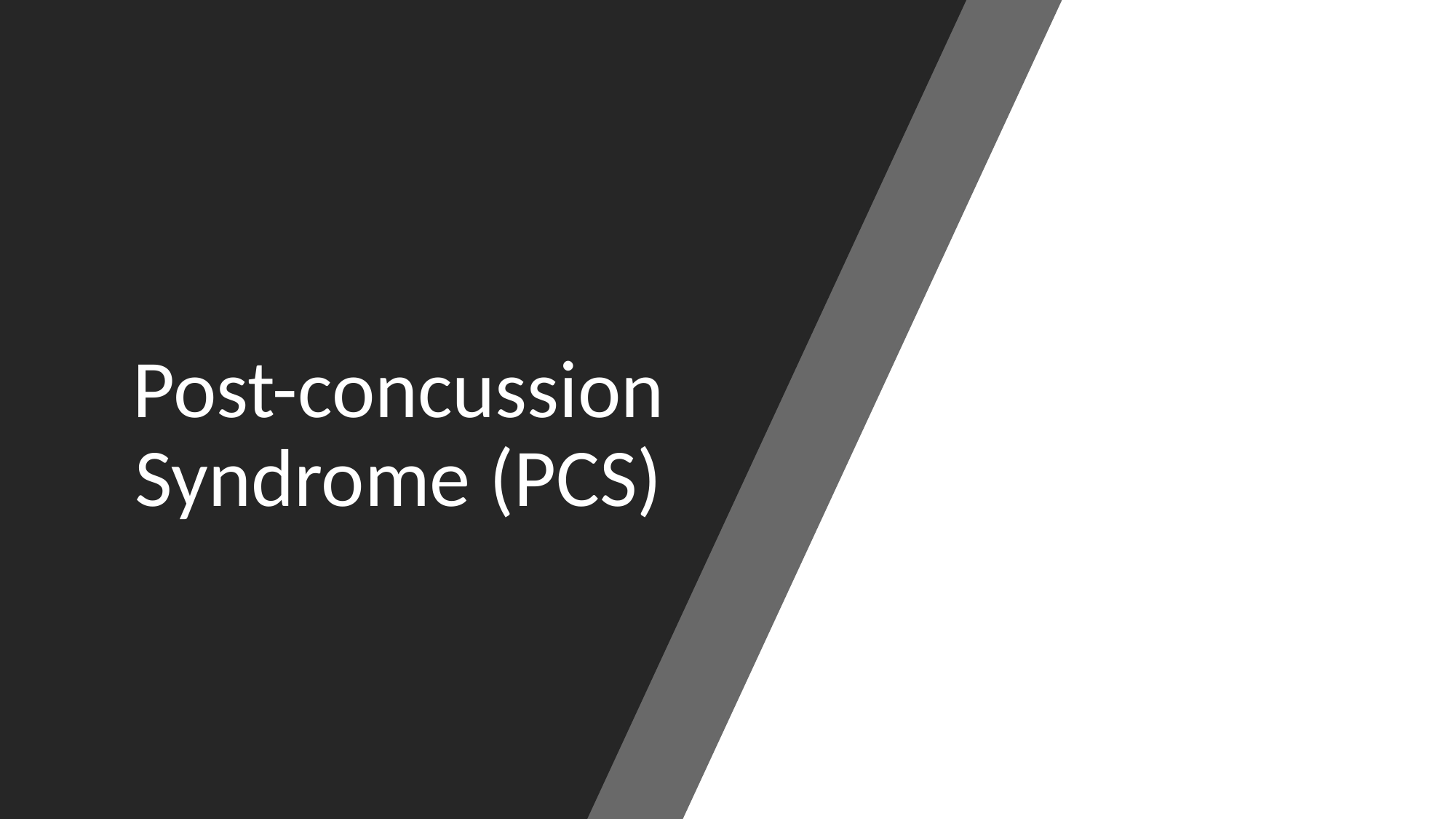

# Post-concussion Syndrome (PCS)

## Slide 35
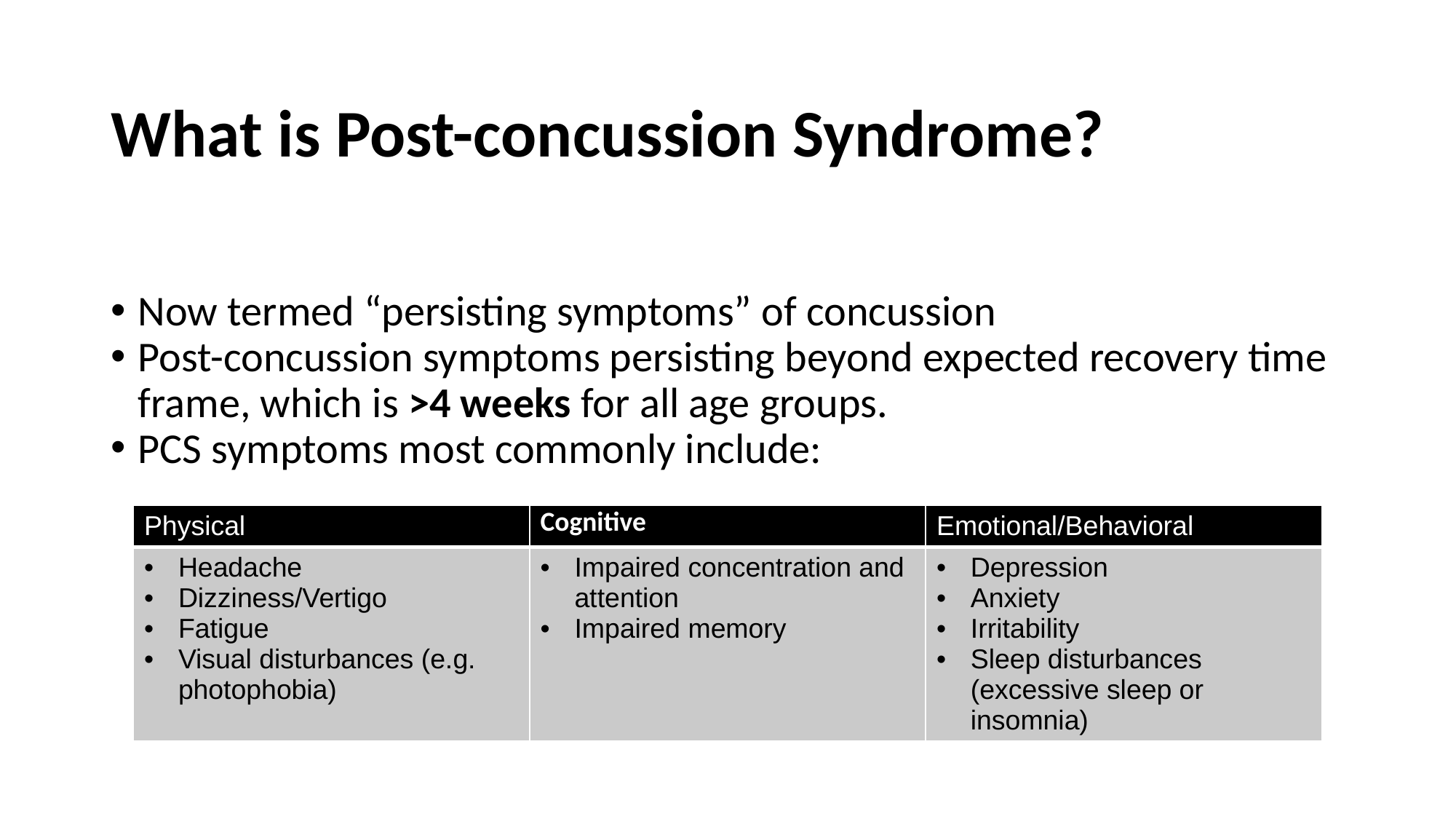

# What is Post-concussion Syndrome?
Now termed “persisting symptoms” of concussion
Post-concussion symptoms persisting beyond expected recovery time frame, which is >4 weeks for all age groups.
PCS symptoms most commonly include:
| Physical | Cognitive | Emotional/Behavioral |
| --- | --- | --- |
| Headache Dizziness/Vertigo Fatigue Visual disturbances (e.g. photophobia) | Impaired concentration and attention Impaired memory | Depression Anxiety Irritability Sleep disturbances (excessive sleep or insomnia) |

## Slide 36
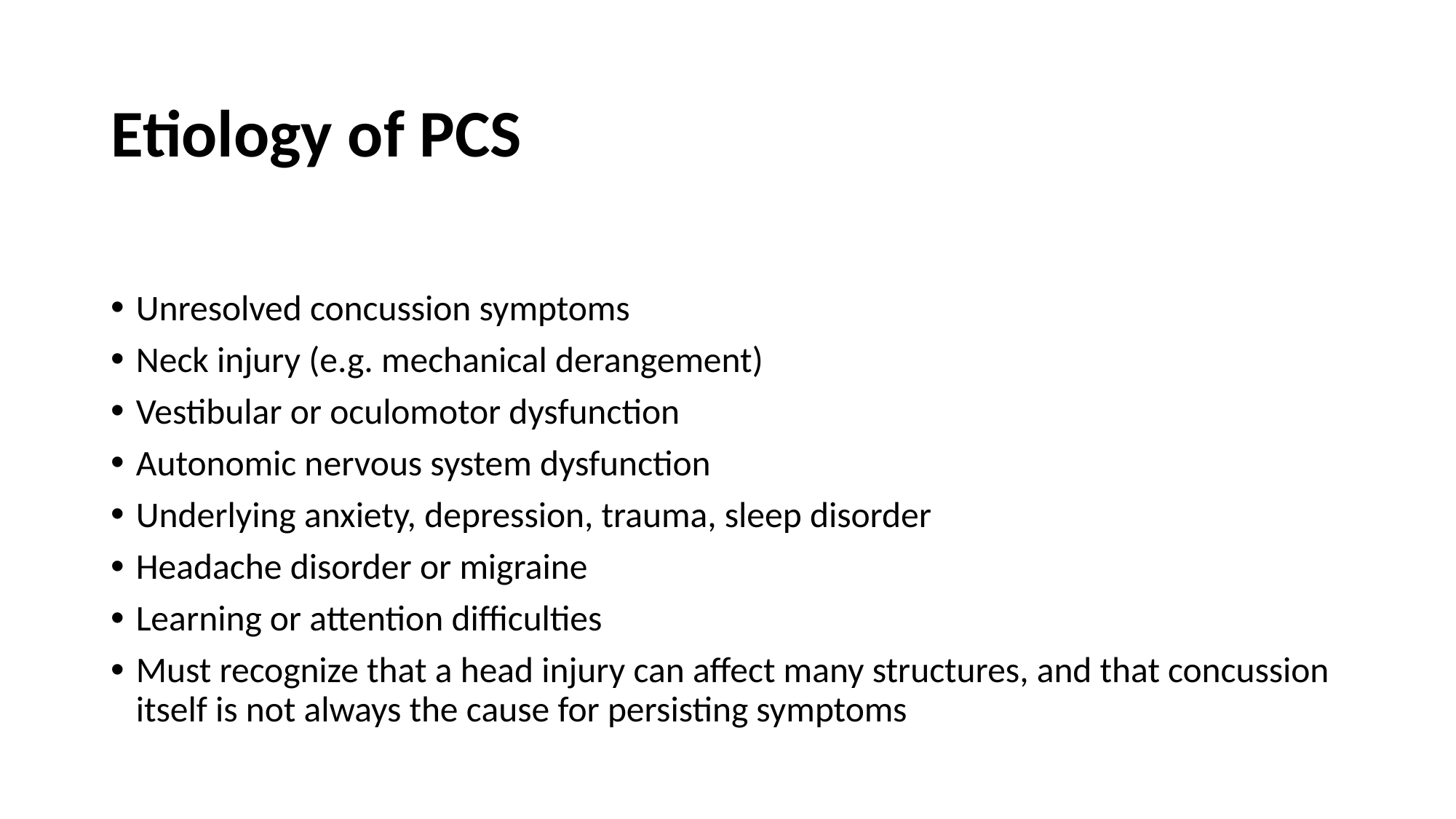

# Etiology of PCS
Unresolved concussion symptoms
Neck injury (e.g. mechanical derangement)
Vestibular or oculomotor dysfunction
Autonomic nervous system dysfunction
Underlying anxiety, depression, trauma, sleep disorder
Headache disorder or migraine
Learning or attention difficulties
Must recognize that a head injury can affect many structures, and that concussion itself is not always the cause for persisting symptoms

## Slide 37
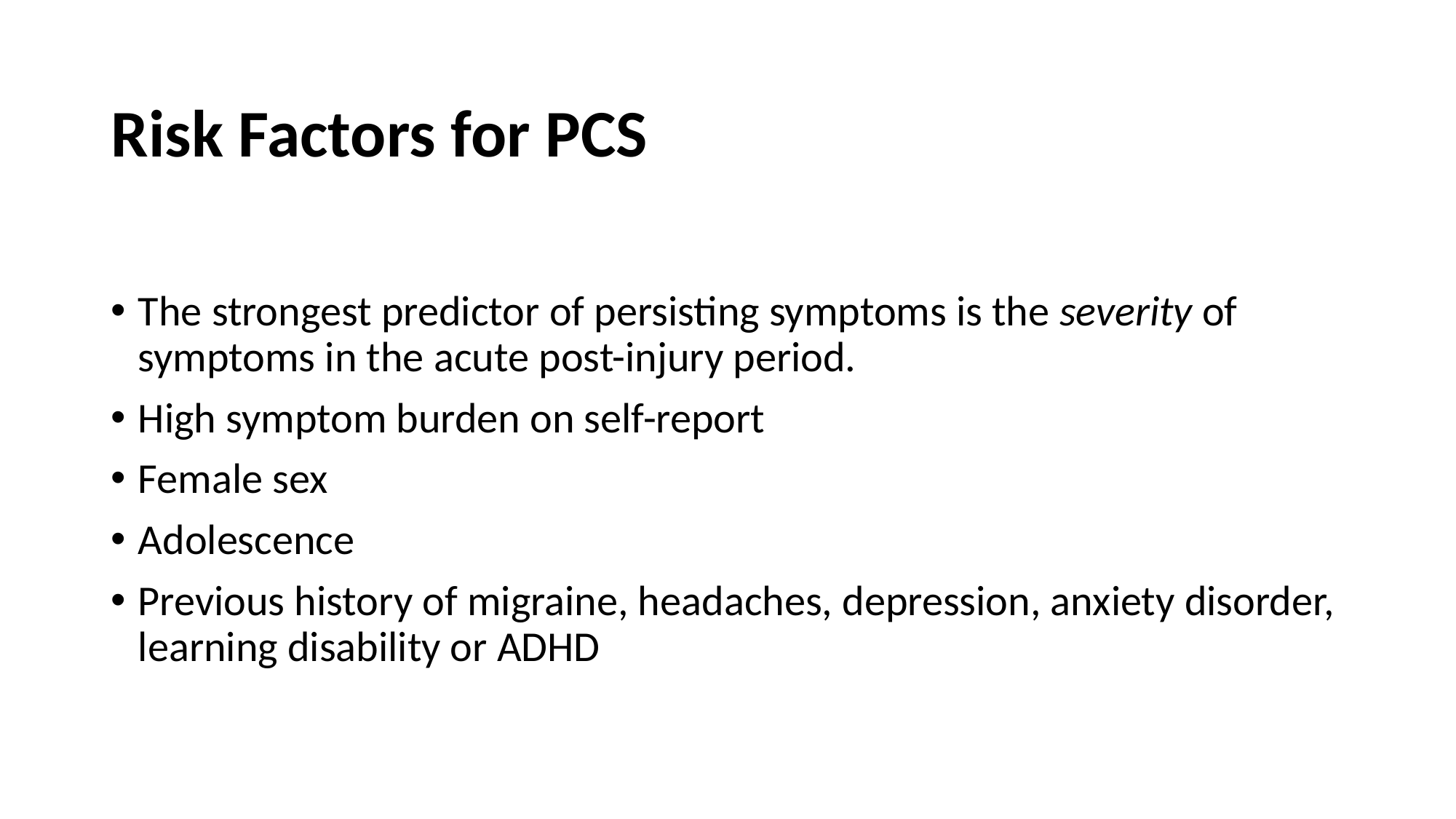

# Risk Factors for PCS
The strongest predictor of persisting symptoms is the severity of symptoms in the acute post-injury period.
High symptom burden on self-report
Female sex
Adolescence
Previous history of migraine, headaches, depression, anxiety disorder, learning disability or ADHD

## Slide 38
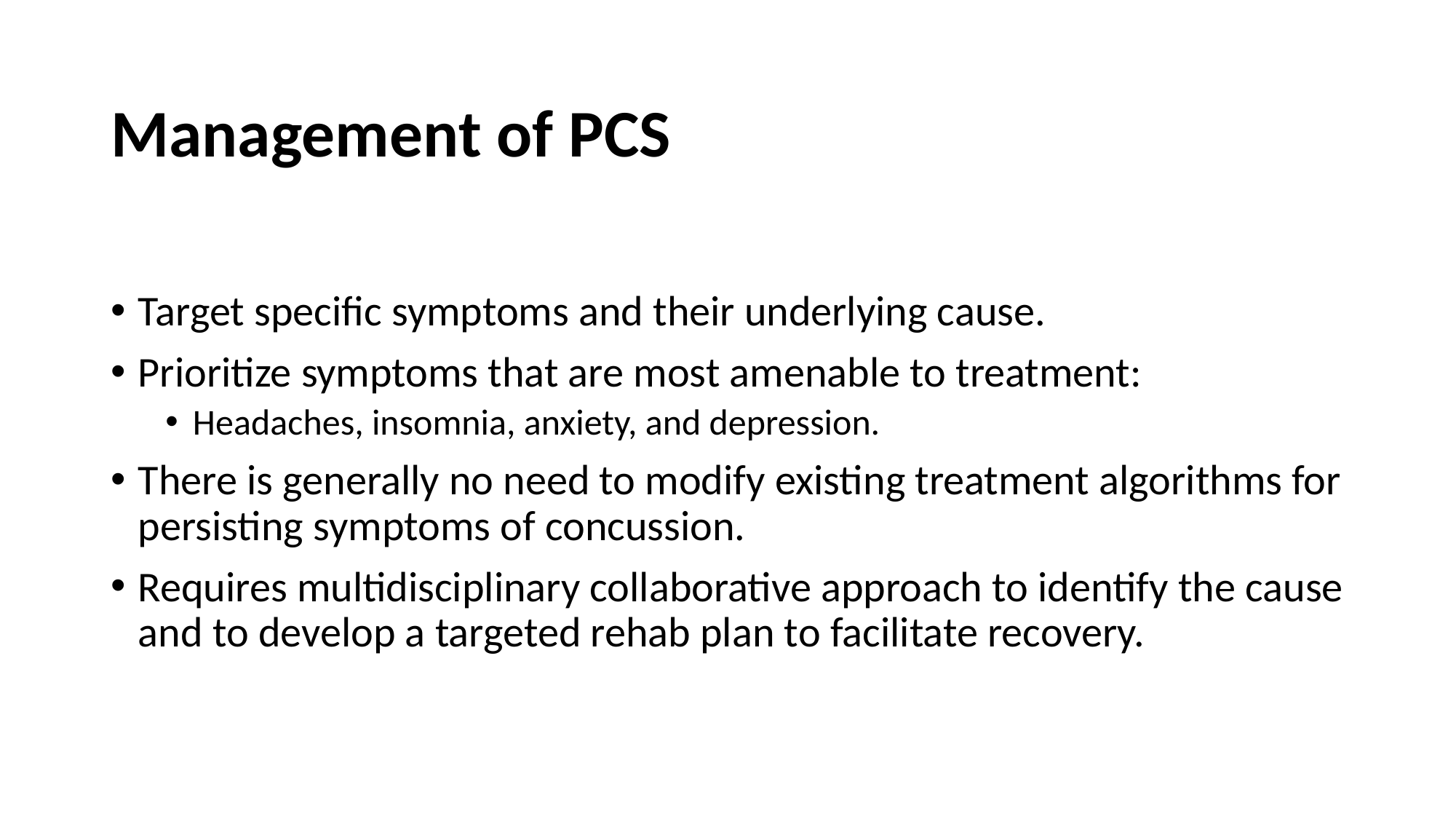

# Management of PCS
Target specific symptoms and their underlying cause.
Prioritize symptoms that are most amenable to treatment:
Headaches, insomnia, anxiety, and depression.
There is generally no need to modify existing treatment algorithms for persisting symptoms of concussion.
Requires multidisciplinary collaborative approach to identify the cause and to develop a targeted rehab plan to facilitate recovery.

## Slide 39
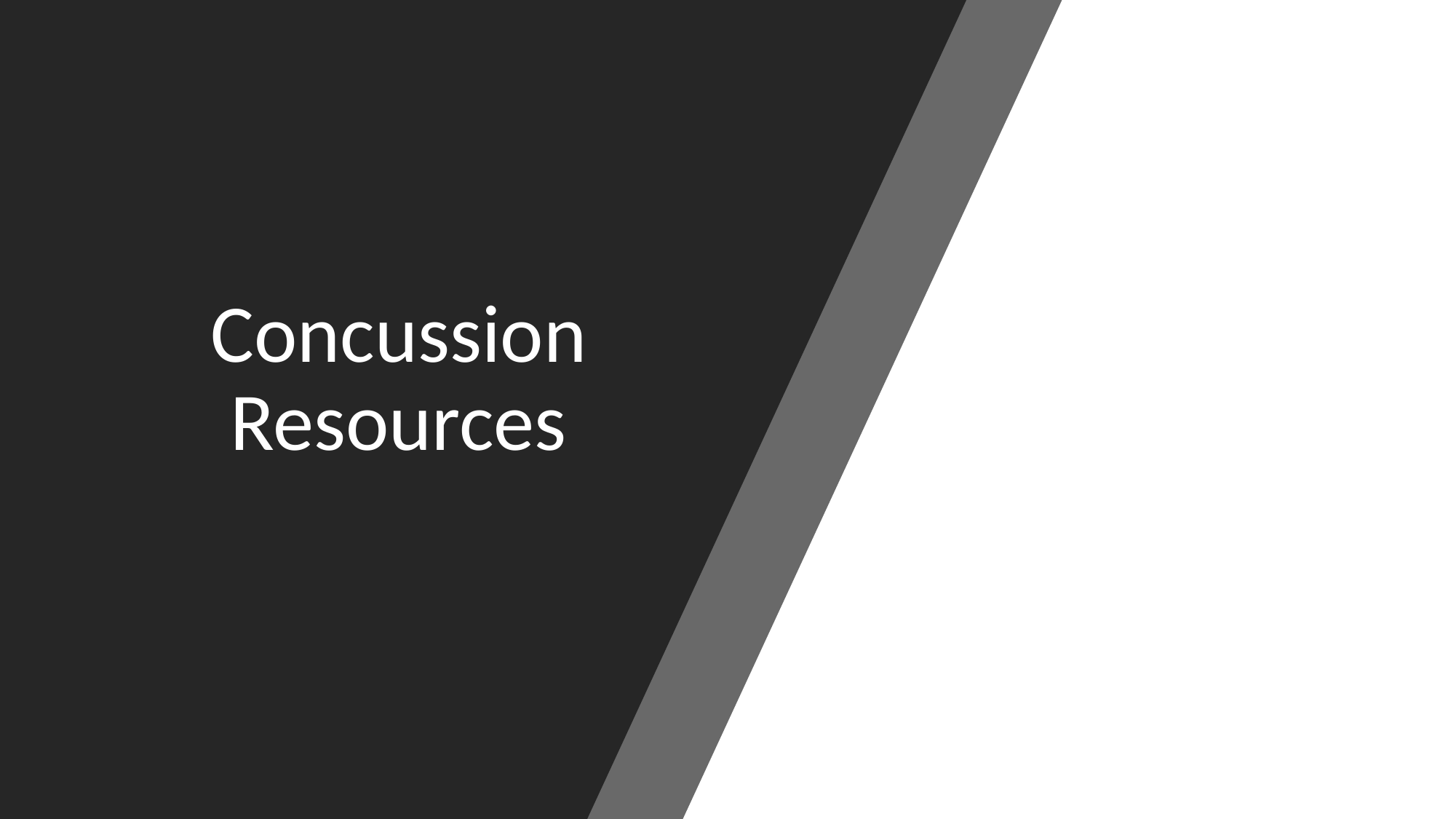

# Concussion Resources

## Slide 40
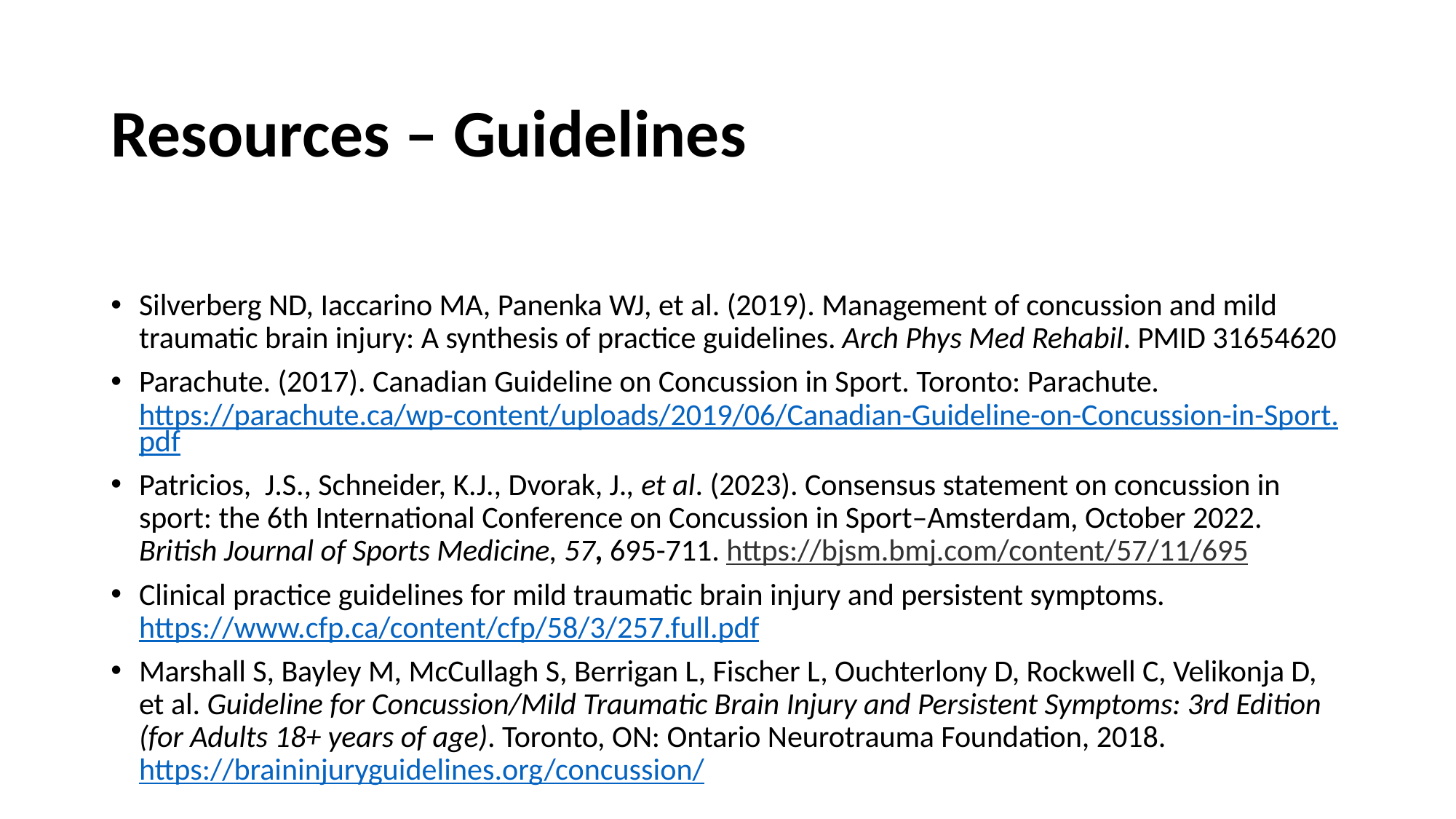

# Resources – Guidelines
Silverberg ND, Iaccarino MA, Panenka WJ, et al. (2019). Management of concussion and mild traumatic brain injury: A synthesis of practice guidelines. Arch Phys Med Rehabil. PMID 31654620
Parachute. (2017). Canadian Guideline on Concussion in Sport. Toronto: Parachute. https://parachute.ca/wp-content/uploads/2019/06/Canadian-Guideline-on-Concussion-in-Sport.pdf
Patricios,  J.S., Schneider, K.J., Dvorak, J., et al. (2023). Consensus statement on concussion in sport: the 6th International Conference on Concussion in Sport–Amsterdam, October 2022. British Journal of Sports Medicine, 57, 695-711. https://bjsm.bmj.com/content/57/11/695
Clinical practice guidelines for mild traumatic brain injury and persistent symptoms. https://www.cfp.ca/content/cfp/58/3/257.full.pdf
Marshall S, Bayley M, McCullagh S, Berrigan L, Fischer L, Ouchterlony D, Rockwell C, Velikonja D, et al. Guideline for Concussion/Mild Traumatic Brain Injury and Persistent Symptoms: 3rd Edition (for Adults 18+ years of age). Toronto, ON: Ontario Neurotrauma Foundation, 2018. https://braininjuryguidelines.org/concussion/

## Slide 41
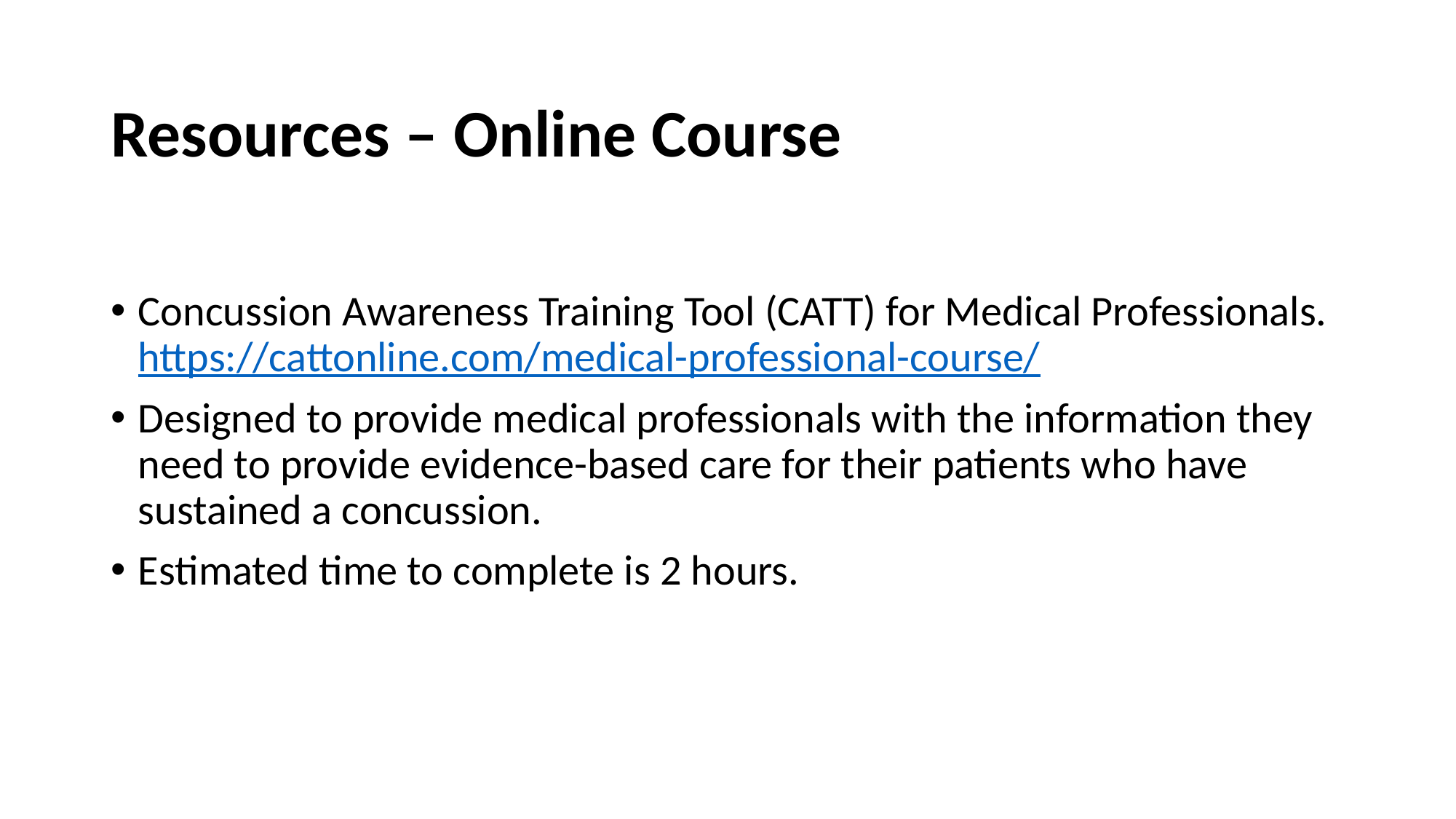

# Resources – Online Course
Concussion Awareness Training Tool (CATT) for Medical Professionals. https://cattonline.com/medical-professional-course/
Designed to provide medical professionals with the information they need to provide evidence-based care for their patients who have sustained a concussion.
Estimated time to complete is 2 hours.

## Slide 42
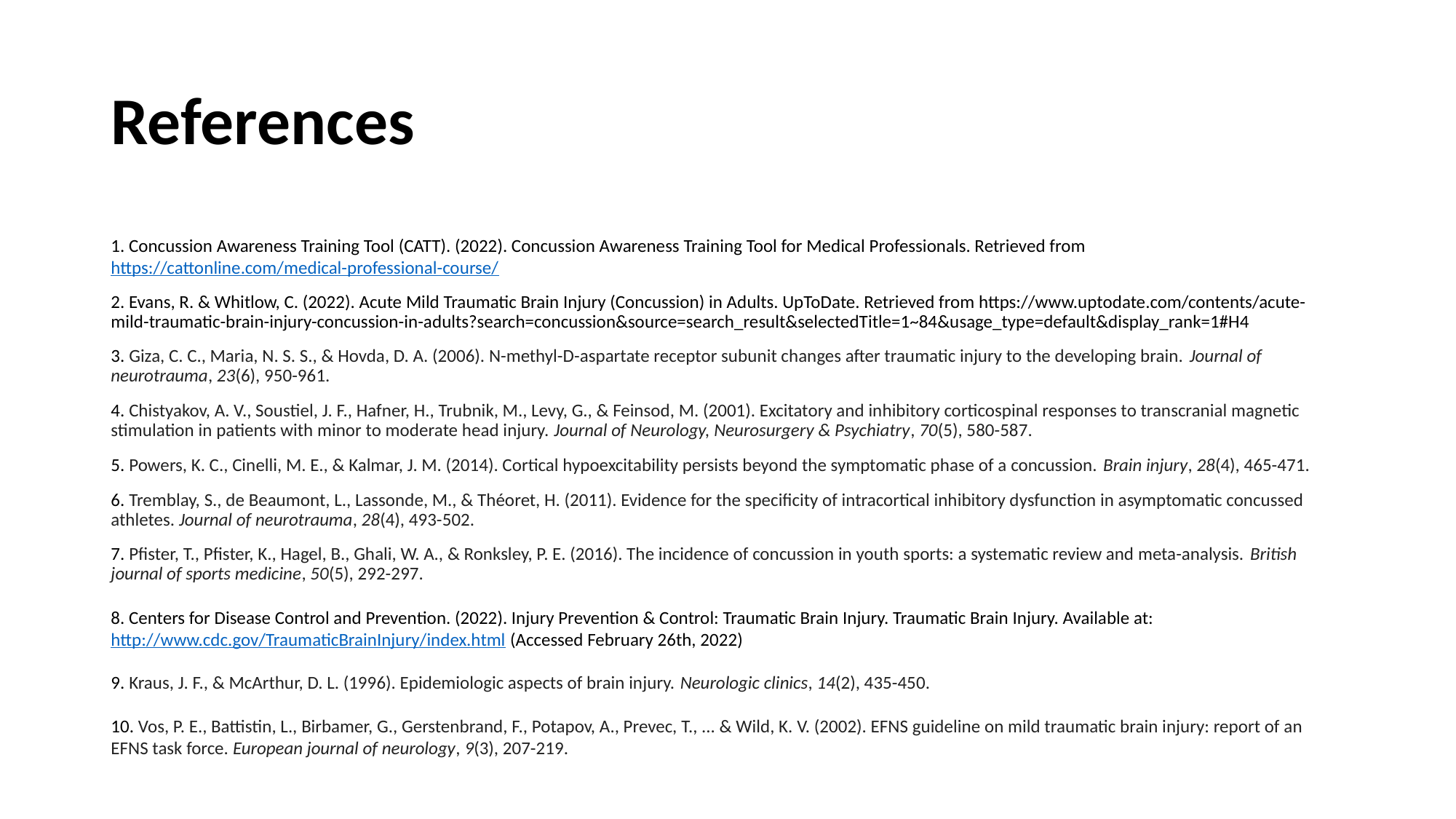

# References
1. Concussion Awareness Training Tool (CATT). (2022). Concussion Awareness Training Tool for Medical Professionals. Retrieved from https://cattonline.com/medical-professional-course/
2. Evans, R. & Whitlow, C. (2022). Acute Mild Traumatic Brain Injury (Concussion) in Adults. UpToDate. Retrieved from https://www.uptodate.com/contents/acute-mild-traumatic-brain-injury-concussion-in-adults?search=concussion&source=search_result&selectedTitle=1~84&usage_type=default&display_rank=1#H4
3. Giza, C. C., Maria, N. S. S., & Hovda, D. A. (2006). N-methyl-D-aspartate receptor subunit changes after traumatic injury to the developing brain. Journal of neurotrauma, 23(6), 950-961.
4. Chistyakov, A. V., Soustiel, J. F., Hafner, H., Trubnik, M., Levy, G., & Feinsod, M. (2001). Excitatory and inhibitory corticospinal responses to transcranial magnetic stimulation in patients with minor to moderate head injury. Journal of Neurology, Neurosurgery & Psychiatry, 70(5), 580-587.
5. Powers, K. C., Cinelli, M. E., & Kalmar, J. M. (2014). Cortical hypoexcitability persists beyond the symptomatic phase of a concussion. Brain injury, 28(4), 465-471.
6. Tremblay, S., de Beaumont, L., Lassonde, M., & Théoret, H. (2011). Evidence for the specificity of intracortical inhibitory dysfunction in asymptomatic concussed athletes. Journal of neurotrauma, 28(4), 493-502.
7. Pfister, T., Pfister, K., Hagel, B., Ghali, W. A., & Ronksley, P. E. (2016). The incidence of concussion in youth sports: a systematic review and meta-analysis. British journal of sports medicine, 50(5), 292-297.
8. Centers for Disease Control and Prevention. (2022). Injury Prevention & Control: Traumatic Brain Injury. Traumatic Brain Injury. Available at: http://www.cdc.gov/TraumaticBrainInjury/index.html (Accessed February 26th, 2022)
9. Kraus, J. F., & McArthur, D. L. (1996). Epidemiologic aspects of brain injury. Neurologic clinics, 14(2), 435-450.
10. Vos, P. E., Battistin, L., Birbamer, G., Gerstenbrand, F., Potapov, A., Prevec, T., ... & Wild, K. V. (2002). EFNS guideline on mild traumatic brain injury: report of an EFNS task force. European journal of neurology, 9(3), 207-219.

## Slide 43
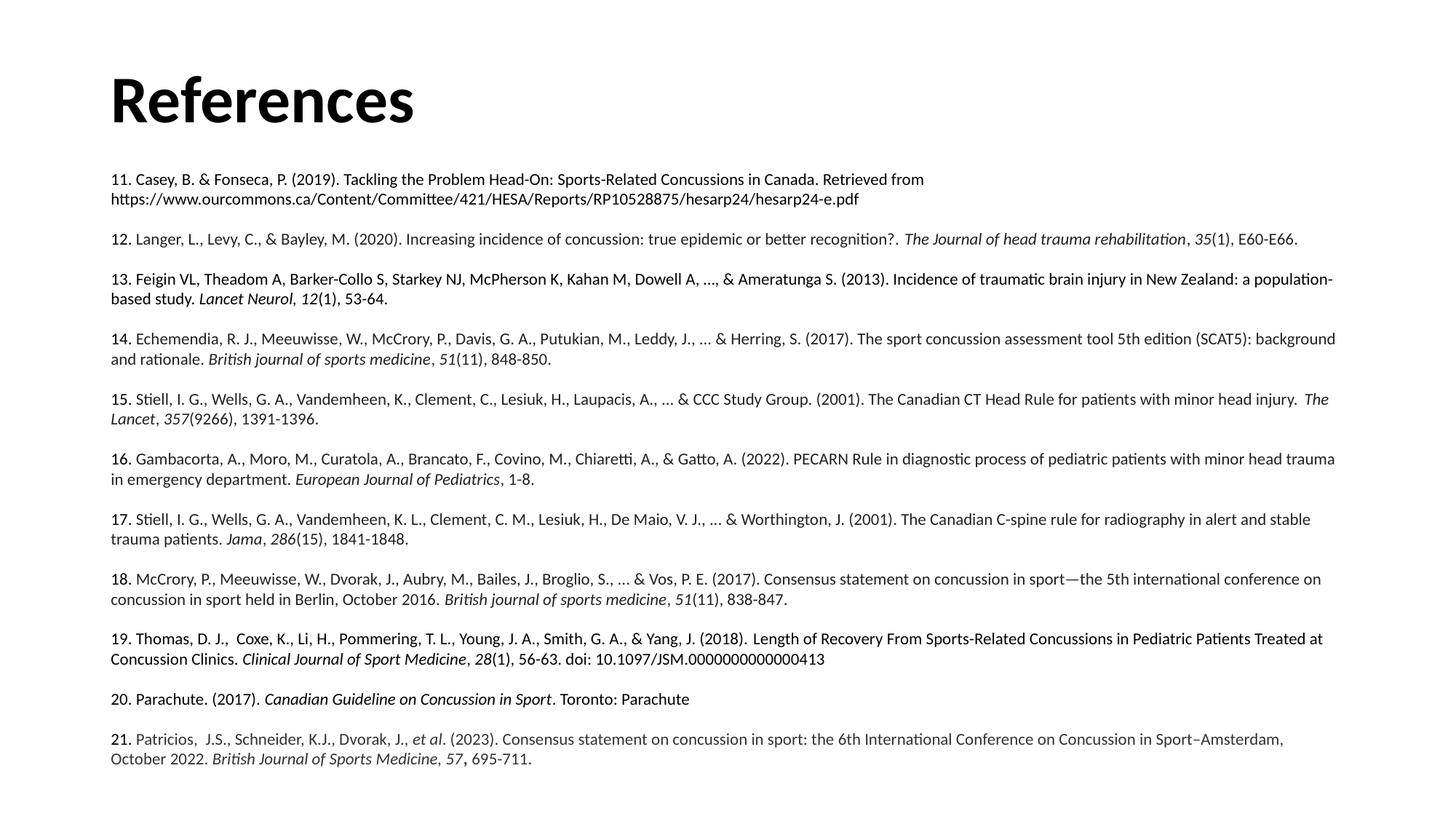

# References
11. Casey, B. & Fonseca, P. (2019). Tackling the Problem Head-On: Sports-Related Concussions in Canada. Retrieved from https://www.ourcommons.ca/Content/Committee/421/HESA/Reports/RP10528875/hesarp24/hesarp24-e.pdf
12. Langer, L., Levy, C., & Bayley, M. (2020). Increasing incidence of concussion: true epidemic or better recognition?. The Journal of head trauma rehabilitation, 35(1), E60-E66.
13. Feigin VL, Theadom A, Barker-Collo S, Starkey NJ, McPherson K, Kahan M, Dowell A, …, & Ameratunga S. (2013). Incidence of traumatic brain injury in New Zealand: a population-based study. Lancet Neurol, 12(1), 53-64.
14. Echemendia, R. J., Meeuwisse, W., McCrory, P., Davis, G. A., Putukian, M., Leddy, J., ... & Herring, S. (2017). The sport concussion assessment tool 5th edition (SCAT5): background and rationale. British journal of sports medicine, 51(11), 848-850.
15. Stiell, I. G., Wells, G. A., Vandemheen, K., Clement, C., Lesiuk, H., Laupacis, A., ... & CCC Study Group. (2001). The Canadian CT Head Rule for patients with minor head injury. The Lancet, 357(9266), 1391-1396.
16. Gambacorta, A., Moro, M., Curatola, A., Brancato, F., Covino, M., Chiaretti, A., & Gatto, A. (2022). PECARN Rule in diagnostic process of pediatric patients with minor head trauma in emergency department. European Journal of Pediatrics, 1-8.
17. Stiell, I. G., Wells, G. A., Vandemheen, K. L., Clement, C. M., Lesiuk, H., De Maio, V. J., ... & Worthington, J. (2001). The Canadian C-spine rule for radiography in alert and stable trauma patients. Jama, 286(15), 1841-1848.
18. McCrory, P., Meeuwisse, W., Dvorak, J., Aubry, M., Bailes, J., Broglio, S., ... & Vos, P. E. (2017). Consensus statement on concussion in sport—the 5th international conference on concussion in sport held in Berlin, October 2016. British journal of sports medicine, 51(11), 838-847.
19. Thomas, D. J., Coxe, K., Li, H., Pommering, T. L., Young, J. A., Smith, G. A., & Yang, J. (2018). Length of Recovery From Sports-Related Concussions in Pediatric Patients Treated at Concussion Clinics. Clinical Journal of Sport Medicine, 28(1), 56-63. doi: 10.1097/JSM.0000000000000413
20. Parachute. (2017). Canadian Guideline on Concussion in Sport. Toronto: Parachute
21. Patricios,  J.S., Schneider, K.J., Dvorak, J., et al. (2023). Consensus statement on concussion in sport: the 6th International Conference on Concussion in Sport–Amsterdam, October 2022. British Journal of Sports Medicine, 57, 695-711.

## Slide 44
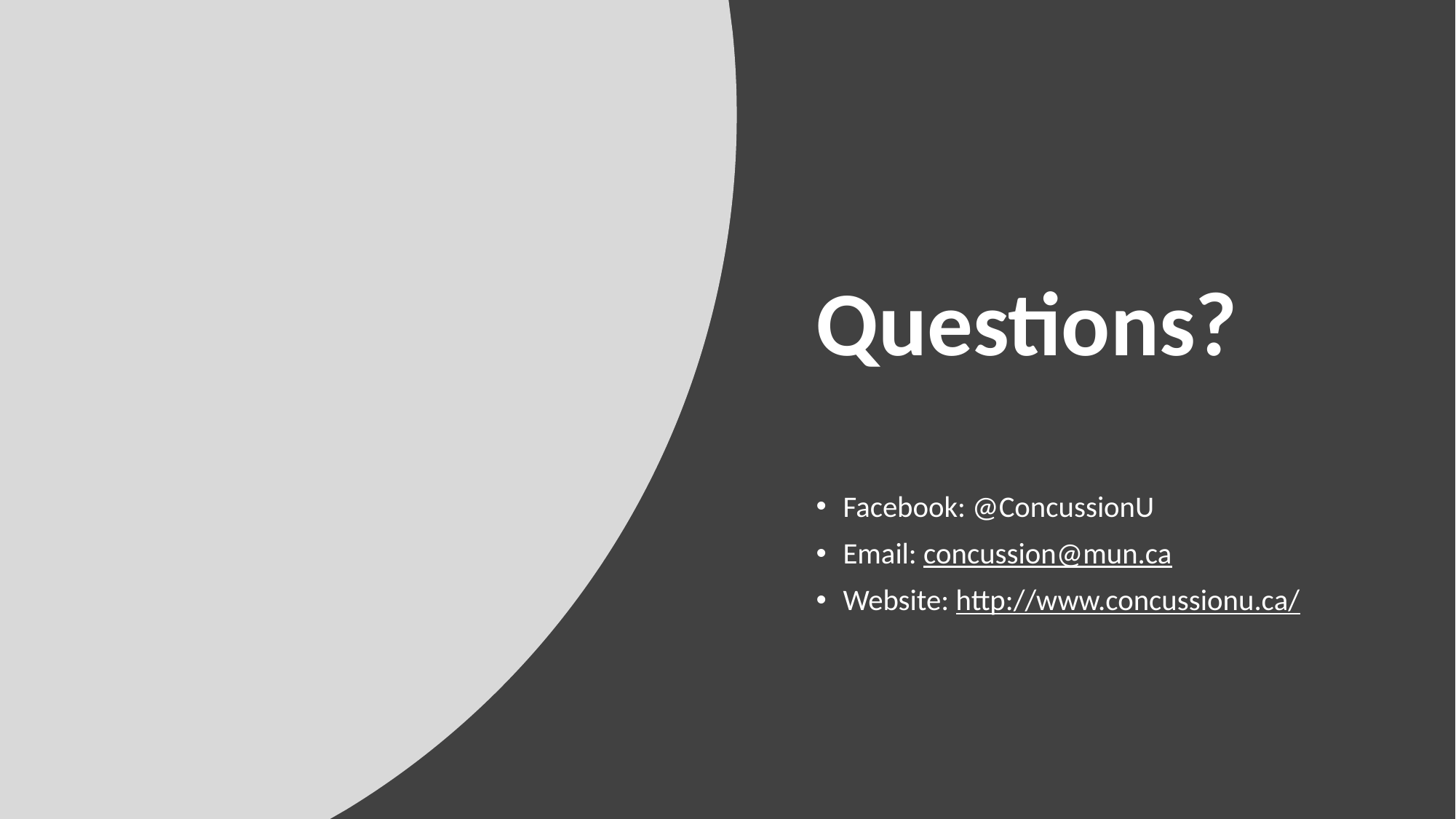

# Questions?
Facebook: @ConcussionU
Email: concussion@mun.ca
Website: http://www.concussionu.ca/
